# Integrating single‐cell and spatial analysis reveals MUC1‐mediated cellular crosstalk in mucinous colorectal adenocarcinoma

**DOI:** 10.1002/ctm2.1701

**Published:** 2024-05-22

**Authors:** Haiyang Zhou, Yiwen Shen, Guangyong Zheng, Beibei Zhang, Anqi Wang, Jing Zhang, Hao Hu, Jiayi Lin, Sanhong Liu, Xin Luan, Weidong Zhang

**Affiliations:** ^1^ Shanghai Frontiers Science Center of TCM Chemical Biology Institute of Interdisciplinary Integrative Medicine Research Shanghai University of Traditional Chinese Medicine Shanghai China; ^2^ Department of Colorectal Surgery Changzheng Hospital Naval Medical University Shanghai China; ^3^ Department of Dermatology Tongren Hospital Shanghai Jiao Tong University School of Medicine Shanghai China; ^4^ Department of Pathology Changzheng Hospital Naval Medical University Shanghai China; ^5^ Department of Pathology Changhai Hospital Naval Medical University Shanghai China; ^6^ Institute of Medicinal Plant Development Chinese Academy of Medical Sciences and Peking Union Medical College Beijing China; ^7^ School of Pharmacy Naval Medical University Shanghai China

**Keywords:** mucinous colorectal adenocarcinoma, scRNA‐seq, spatial transcriptomics, tumour microenvironment

## Abstract

**Background:**

Mucinous colorectal adenocarcinoma (MCA) is a distinct subtype of colorectal cancer (CRC) with the most aggressive pattern, but effective treatment of MCA remains a challenge due to its vague pathological characteristics. An in‐depth understanding of transcriptional dynamics at the cellular level is critical for developing specialised MCA treatment strategies.

**Methods:**

We integrated single‐cell RNA sequencing and spatial transcriptomics data to systematically profile the MCA tumor microenvironment (TME), particularly the interactome of stromal and immune cells. In addition, a three‐dimensional bioprinting technique, canonical ex vivo co‐culture system, and immunofluorescence staining were further applied to validate the cellular communication networks within the TME.

**Results:**

This study identified the crucial intercellular interactions that engaged in MCA pathogenesis. We found the increased infiltration of *FGF7* ^+^/*THBS1* ^+^ myofibroblasts in MCA tissues with decreased expression of genes associated with leukocyte‐mediated immunity and T cell activation, suggesting a crucial role of these cells in regulating the immunosuppressive TME. In addition, *MS4A4A* ^+^ macrophages that exhibit M2‐phenotype were enriched in the tumoral niche and high expression of *MS4A4A* ^+^ was associated with poor prognosis in the cohort data. The ligand‐receptor‐based intercellular communication analysis revealed the tight interaction of *MUC1* ^+^ malignant cells and *ZEB1* ^+^ endothelial cells, providing mechanistic information for MCA angiogenesis and molecular targets for subsequent translational applications.

**Conclusions:**

Our study provides novel insights into communications among tumour cells with stromal and immune cells that are significantly enriched in the TME during MCA progression, presenting potential prognostic biomarkers and therapeutic strategies for MCA.

**Key points:**

- Tumour microenvironment profiling of MCA is developed.
- *MUC1* ^+^ tumour cells interplay with *FGF7* ^+^/*THBS1* ^+^ myofibroblasts to promote MCA development.
- *MS4A4A* ^+^ macrophages exhibit M2 phenotype in MCA.
- *ZEB1* ^+^ endotheliocytes engage in EndMT process in MCA.

## Graphical abstract

(1) Tumor microenvironment profiling of MCA is developed. (2) *MUC1* ^+^ tumour cells interplay with *FGF7* ^+^/*THBS1* ^+^ myofibroblasts to promote MCA development. (3) *MS4A4A* ^+^ macrophages exhibit M2 phenotype in MCA. (4) *ZEB1* ^+^ endotheliocytes engage in the EndMT process in MCA.

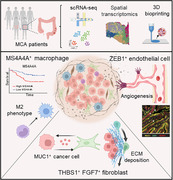

## 1 INTRODUCTION

Mucinous colorectal adenocarcinoma (MCA) is a distinct subtype of colorectal cancer (CRC) and is found in 10−20% of patients with CRC.^1^, ^2^ Compared with common colorectal adenocarcinoma, MCA has entirely distinct clinical, histopathological, and molecular characteristics, as well as an aberrant and aggressive metastatic pattern associated with inadequate response to treatment and worse prognosis.^3^ From a clinical perspective, MCA is associated with reduced rates of pathological complete response and tumor downstaging after neoadjuvant chemoradiotherapy compared with non‐mucinous rectal cancer.^4^, ^5^ Although immune checkpoint blockade therapy has shown significant efficacy in various solid tumors, MCA patients exhibited a poorer response to PD‐L1 inhibitors than patients lacking the mucinous component.^6^ From a histological and molecular perspective, MCA tumor is characterised by extracellular mucin in more than 50% of the lesion.^7^ The overexpression of mucin proteins, such as MUC1, MUC2, and MUC5AC in MCA tumors that form a mucous layer protects tumour cells against immunotherapy.^8^, ^9^ Previous studies have indicated that patients with high expression of MUC1 exhibited significantly worse disease‐free survival, and patients with high MUC2 expression showed worse overall survival.^10^ It has also been reported that the MUC1 N‐terminal subunit (MUC1‐N) functions as a putative receptor that mediates diverse signaling pathways to promote tumor progression.^11^

To date, the unique mucinous histology of MCA is not included in the determination of treatment options, and no tailored and practical approach is approved for MCA patients.^1^, ^2^ CRC has a diverse cellular microenvironment whereby heterotypic interactions are essential in defining its aetiology and response to treatment. Therefore, it is necessary to understand the mechanism of cellular and molecular characteristics in the MCA tumour microenvironment (TME) to determine the potential efficient interventions. Single‐cell RNA‐sequencing (scRNA‐seq) offers a remarkable new platform to delineate the complex TME landscape and the interactions among diverse cellular components contributing to cancer progression.^12^, ^13^ Until now, few reports regarding MCA have exploited scRNA‐seq analysis to characterise the MCA atlas at the single‐cell resolution and depict the cell–cell interactions in the MCA environment.

In the present study, we constructed a single‐cell map of malignant MCA cells by integrating scRNA‐seq data, cell co‐culture models, spatial transcriptomics, and clinical information from the TCGA cohort and Chinese cohort. Then, the transcriptional profiles, regulatory pathways, and intercellular communications were explored to decipher key cellular populations in tumorigenesis and elucidate the pathological mechanism of MCA, which could deepen our understanding of the immunobiology of MCA and might play an instructive role in chemotherapy and immunotherapy for MCA.

## 2 RESULTS

### 2.1 A single‐cell transcriptomic landscape of paired human MCA tissues and normal colon tissues

**FIGURE 1:**
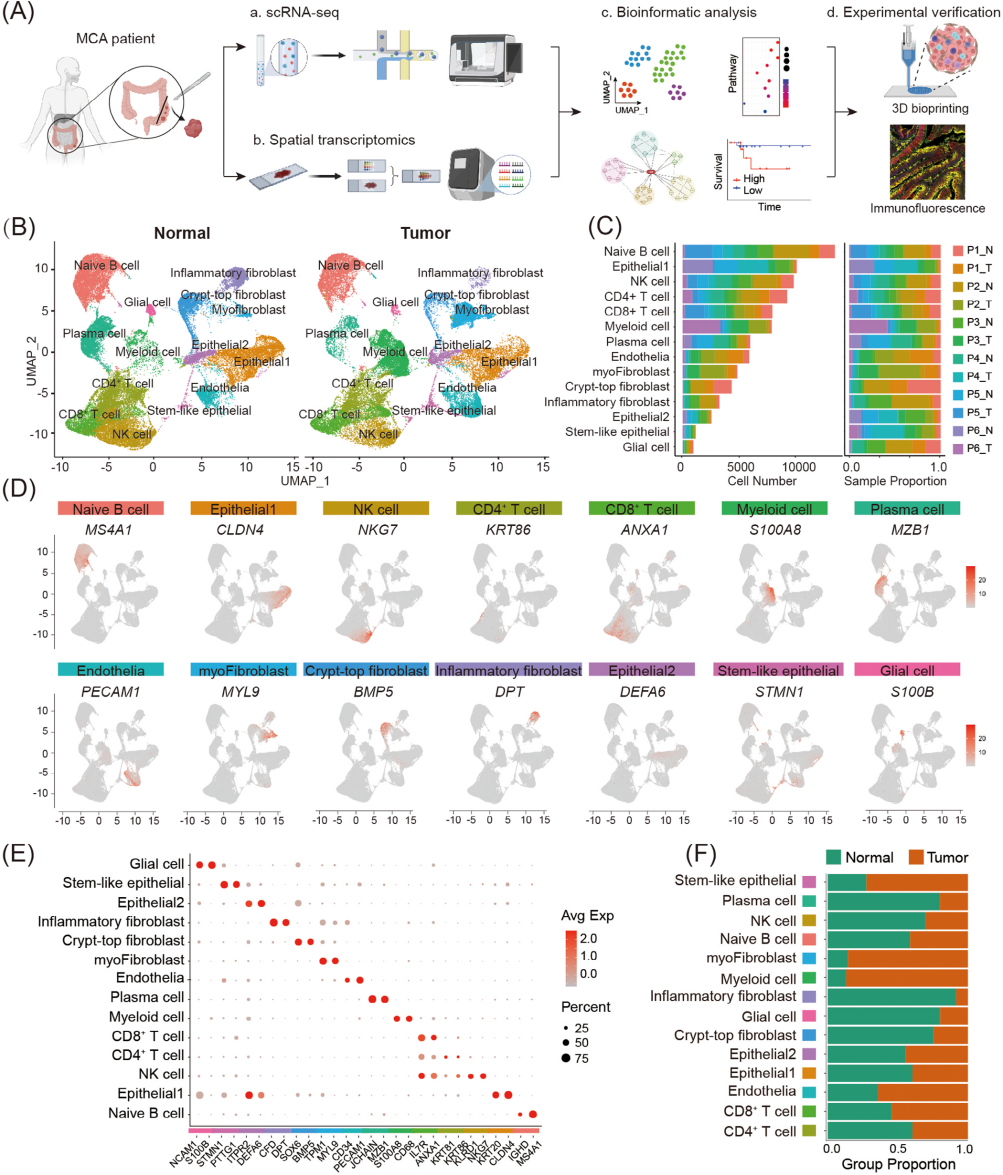
Cell landscape of paired human MCA tissues and normal colon tissues. (A) Graphic overview of the presented study design. (B) UMAP plots of 40 424 and 48 081 cells originating from tumour and normal tissues of six MCA patients. Fourteen cell clusters were identified. (C) Bar plot showing distributions (left) and proportions (right) of cells derived from every sample for each cell cluster. (D) Expressive distribution of signature genes in overall cells for each cell cluster. (E) Dot plot showing the expression profile of signature genes in each cell cluster. (F) Cell proportions of the normal and tumour groups in each cell cluster.

In the present study, a strategy of single‐cell RNA sequencing (scRNA‐seq) combined with spatial transcriptomic and cell co‐culture assays was applied to explore the cellular composition and cell communication of mucinous colorectal adenocarcinoma (Figure 1A). Six paired human MCA tissues and adjacent normal colon tissues (left‐sided colon, *n* = 2; right‐sided colon, *n* = 2; rectum, *n* = 2;) were first collected by surgical operation (clinical information of these six MCA patients is shown in Table S1). Samples of MCA and normal tissues were then processed with 3′‐end single‐cell RNA sequencing to present a cellular transcriptomic landscape. Quality control of sequencing was conducted to filter dead cells. Thus, a total of 88 505 cell transcriptomes were obtained, of which 40 424 and 48 081 cells originated from tumour and normal tissues, respectively (Figure 1B; Figure S1). Afterwards, normalisation for different samples was performed to correct the batch effect. Highly variable expression genes across sequencing cells of samples were identified and used as features of principal component analysis (PCA) to build a dimensionality reduction space. Then, the UMAP algorithm was used to classify the sequencing cells, and 14 cell clusters were presented. The 14 cell clusters included naïve B cells identified by the expression of *IGHD* and *MS4A1* (*n *= 13 460), epithelial cells (subset 1) marked by *CLDN4* and *KRT20* expression (*n* = 10 220), NK cells expressed *NKG7* and *KLRD1* positively (*n* = 9952), CD4^+^ T cells characterised by expression of *KRT81* and *KRT86* (*n* = 9362), CD8^+^ T cells identified by *IL7R* and *ANXA1* expression (*n* = 8030), myeloid cells expressed *S100A8* and *CD68* positively (*n* = 7977), plasma cells marked by expression of *JCHAIN* and *MZB1* (*n* = 6048), endothelial cells captured by expression of *CD34* and *PECAM1* (*n* = 5965), myofibroblasts identified by the expression of *TPM1* and *MYL9* (*n* = 4901), crypt‐top fibroblasts marked by *SOX6* and *BMP5* expression (*n* = 4392), inflammatory fibroblasts expressed *CFD* and *DPT* remarkably (*n* = 3305), epithelial cells (subset 2) marked by expression of *ITPR2* and *DEFA6* (*n* = 2568), stem‐like epithelial cells characterised by *PTTG1* and *STMN1* expression (*n* = 1169), and enteric glial cells expressed *NCAM1* and *S100B* highly (*n* = 958) (Figure 1C, D, E). The fraction of each cell cluster in every sample was variable, which might reflect each patient's different stages of tumour progression (Figure 1C). Meanwhile, there was a noticeable ratio difference between the tumour and normal sample groups in fourteen cell clusters, which possibly reflected the tumour microenvironment and might result from cancer cell infiltration (Figure 1F).

### 2.2 Dynamic changes in cell clusters and communications between tumour and normal tissues reveal key cellular populations in tumour progression

**FIGURE 2:**
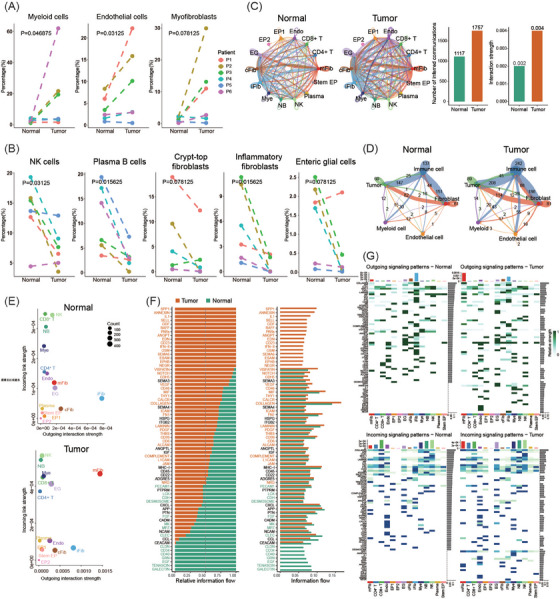
Dynamic changes in cell populations and communication in the tumour microenvironment. (A and B) Percentage comparison between normal and tumour groups in elevated (A) and decreased (B) cell clusters with a significant difference. (C) Communication comparison between normal and tumour groups among cell clusters. (D) Communication comparison between normal and tumour groups among immune, fibroblast, myeloid, endothelium, and tumour modules. (E) Strength of links (including incoming and outgoing links) among cell clusters. (F) Information flow comparison between normal and tumour groups in signalling pathways. (G) Comparison of ligand‐receptor pair interactions between the normal and tumour groups at the pathway level among cell clusters.

To investigate the dynamic change in cellular populations for MCA, we compared the percentage of each cell cluster between the tumor and normal groups (Figure 2A, B). Except for stem‐like epithelial cells (*p *= 0.078, one‐sided Wilcoxon test), three cellular populations in the tumor group showed increased percentages compared to normal ones. There was an elevated percentage of myeloid cells in the tumor group (*p *= 0.047, one‐sided Wilcoxon test), which hinted that these cells might be recruited in the tumoral niche to help proliferation and invasion.^14^ Endothelial cells also showed a higher percentage in tumor samples (*p *= 0.031, one‐sided Wilcoxon test), which suggested these cells might play an essential role in promoting MCA progression.^15^, ^16^ For myofibroblasts, an increased percentage was observed in tumor samples with weak statistical significance (*p *= 0.078, one‐sided Wilcoxon test), indicating that myofibroblasts might act as cancer‐associated fibroblasts to promote tumor progression.^17^ For NK cells and plasma B cells, a decreased percentage was shown in the tumor group (*p *= 0.031 and *p *= 0.016, one‐sided Wilcoxon test), which suggested that immunosuppression existed in the tumor microenvironment.^18^ A lower percentage was detected in crypt‐top fibroblasts and inflammatory fibroblasts for tumor samples (*p *= 0.078 and *p *= 0.016, one‐sided Wilcoxon test), which implied that crypt‐top fibroblasts and inflammatory fibroblasts might be transformed into cancer‐associated fibroblasts; thus, a reduced population was observed in the tumoral niche.^19^ There was also a decreased percentage of enteric glial cells in the tumor group (*p *= 0.078, one‐sided Wilcoxon test) since they might be tamed by tumor cells and transformed into cancer stem cells.^20^ For other cell clusters (including naïve B cells, CD4^+^ T cells, CD8^+^ T cells, epithelial cell subset 1, and epithelial cell subset 2, the change in the percentage of these cell clusters is shown in Figure S2A), no significant difference was observed when comparing the tumor and normal groups.

In addition to the percentage change in cellular populations for MCA, the dynamics of cell communication for MCA were also investigated. Compared with the normal group, more and much stronger communication was detected in the tumour group (Figure 2C; Figure S2B). To highlight the dynamics of communication across cell clusters, interactions among the immune module (including naïve B cells, NK cells, CD4^+^ T cells, CD8^+^ T cells, and plasma cell populations), tumour module (including stem‐like epithelial cells, epithelial cell subset 1, and subset 2), fibroblast module (including crypt‐top fibroblasts, myofibroblasts, and inflammatory fibroblasts), myeloid module (including myeloid cells), and endothelial module (including endothelial cells) were detected. Compared with the normal group, communications in the tumour group among all modules showed a remarkable increase, which suggested that immune, myeloid, fibroblast, and endothelial populations might play essential roles in tumour progression (Figure 2D; Figure S2C). Moreover, the strength of interactions (including incoming and outgoing links) among cell clusters was analyzed. Myofibroblasts and myeloid cells showed higher strength in both incoming and outgoing links with other cell populations, especially with tumour populations. For endothelial cells, there was a remarkable increase in outgoing links and a moderate decrease in incoming links. These findings indicated that myofibroblasts, myeloid cells, and endothelial cells were key cellular populations in the development of MCA (Figure 2E).

In addition, communications among cell populations at the signal pathway level were predicted based on ligand‐receptor pair interactions of pathways. Compared with the normal group, the tumour group showed much more information flow and closer association in the following signalling pathways defined in CellChatDB: SPP1, ANNEXIN, IL1, SELL, GDF, BAFF, PARS, ANGPT, EDN, CD23, INF‐II, OSM, SEMA6, ESAM, EPHB, and NEGR (Figure 2F). In addition, both the incoming and outgoing signal patterns of ligand‐receptor pair interactions of pathways among cell clusters were inspected. Inspection results showed that the tumour‐associated pathways were primarily involved in myofibroblasts, inflammatory fibroblasts, endothelial cells, and myeloid cells (Figure 2G; Figure S3); significant differences in ligand‐receptor pair interactions in signalling pathways among cell clusters between the tumour and normal groups are shown in Figure S2D–L. Taken together, the results of dynamic changes in percentages and communications for cell clusters suggested that myeloid cells, endothelial cells, and myofibroblasts were elevated populations in the tumour environment, and they presented strong communicative links with cancer cells, which contributed to morphogenesis of the tumoural niche in MCA.

### 2.3 Single‐cell transcriptomic profile unveils the expression signatures and signalling pathways of elevated cell clusters associated with tumour progression

It is well known that the mucin protein family is highly expressed in mucinous colorectal adenocarcinoma,^2^ so the expression of secreted mucins (including *MUC5AC*, *MUC5B*, *MUC6*) and transmembrane mucins (including *MUC1*, *MUC3A*, *MUC4*, *MUC12*, *MUC13*, *MUC15*, *MUC16*, *MUC17*, *MUC20*) was detected in each cell cluster. It was found that both transmembrane mucins and secreted mucins showed high expression in epithelial populations, including stem‐like epithelial cells, epithelial cell subset 1, and epithelial cell subset 2 (Figure S4A, B). Notably, MUC1 and MUC4 are highly expressed in stem‐like epithelial cells and epithelial cell subset 1. It is noteworthy that MUC1 has been linked to tumorigenesis and the progression of MCA.^21^ In addition to mucins, the expression of cytokines (including IL2, IL10, IL15, IFNG, TNF, TNFRSF1A, TNFRSF1B, IL6, IL1A, IL1B, IL1R1, IL1R2, IGFB1, TGFBR1, and TGFBR2) and chemokine receptors (CCR1, CCR2, CCR3, CCR4, CCR5, CCR7, CXCL8, CXCR1, CXCR2, CXCR4, CSF1, and CSF1R) in each cell cluster was also detected (Figure S4C, D) since they are essential factors of the tumour microenvironment and are closely correlated with tumorigenesis.^22^ Notably, except for immune populations (including naïve B cells, NK cells, CD4^+^ T cells, CD8^+^ T cells, and plasma cells), these cytokines and chemokine receptors were highly expressed in myeloid cells, which implied that myeloid cells might play a significant role in tumorigenesis. To further analyze the malignant feature of MCA cells, enrichment analysis was performed to determine the signalling pathways that drive MCA development. Interestingly, we found that PI3K→AKT and MEK→ERK signalling were highly enriched in MCA tumour samples, particularly in stem‐like epithelial cells rather than other cell types (Figure S4E, F; Table S2). The results indicated that PI3K→AKT and MEK→ERK signalling promoted the malignant phenotypes of epithelial cells and facilitated MCA progression.

To identify more critical genes involved in tumour progression, analysis of differentially expressed genes (DEGs) between the tumour and normal groups was carried out for elevated cell populations in the tumour environment (i.e., myeloid cells, endothelial cells, and myofibroblasts). Then, GO enrichment and pathway overrepresented analysis were performed to unveil biological processes and signalling pathways affected by MCA development. It is worth noting that in the tumoural niche, highly expressed genes in myeloid cells were mainly correlated with chemokine responding, cell chemotaxis, leukocyte differentiation, activation, and migration. In contrast, the genes with low expression were associated with embryonic hemopoiesis, microvillus organisation, and some physiological behaviours of somatic cells, such as fatty acid biosynthesis, cell maturation and maintenance, exocytosis, and compound transportation (Figure S5A, D, E). These results indicated that myeloid cells might play a complex role in the progression of MCA. On the one hand, myeloid cells were hijacked by tumour cells and thus secreted a great number of chemokines, which played a vital role in tumour progression and metastasis.^23^ On the other hand, myeloid cells secrete important cytokines and maintain the normal functions of somatic cells.^24^ For endothelial cells, it was found that highly expressed genes in tumour conditions were primarily involved in the process of extracellular matrix (ECM) organisation, cell‐substrate adhesion, and epithelial‐to‐mesenchymal transition (EMT) (Figure S5B, F, G). The process of cell adhesion is regarded as a crucial event in tumour invasion and metastasis, which is influenced by the process of extracellular matrix deposition and crosslinking.^25^ The EMT process is associated with the cell adhesion property of tumour cells and is thought to be a significant route of tumour development and progression.^26^ Simultaneously, downregulated genes in tumour conditions were mainly correlated with cell activation, leukocyte‐mediated immunity, defence response to the virus, and immune response, suggesting that endothelia's immune and virus defence functions were dysregulated and immunity was modulated in the tumoural niche. For myofibroblasts, upregulated genes in the tumour group were primarily correlated with extracellular matrix organisation, collagen metabolic process, glycoprotein biosynthetic and metabolic process, aminoglycan and glycosaminoglycan metabolic process, and regulation of apoptotic signalling pathway (Figure S5C, H, I). The downregulated genes were closely correlated with muscle system processes, calcium ion transportation, and cation homeostasis. These findings suggest that myofibroblasts present more hallmarks of cancer‐associated fibroblasts (CAFs) than traditional fibroblasts in the tumoural niche. Myofibroblasts might participate in the invasion process of tumour cells since upregulated genes were highly associated with ECM organisation and corresponding signalling pathways, which are remarkably correlated with tumour invasion.^27^ Moreover, myofibroblasts play a crucial role in tumour cell migration, infiltration, and invasion by regulating collagen metabolism, which affects the tumour microenvironment and cancer cell fibrosis and thus dramatically influences tumour biology and therapeutic response.^28^ In summary, these results suggested that cytokine and chemokine association pathways, ECM correlation pathways, EMT pathways, and collagen metabolic pathways involving myeloid cells, endothelial cells, and myofibroblasts were correlated with the formation of tumour pre‐metastatic niche and infiltration of cancer cells.

### 2.4 Myofibroblasts act as cancer‐associated fibroblasts and promote tumour cell migration and invasion

**FIGURE 3:**
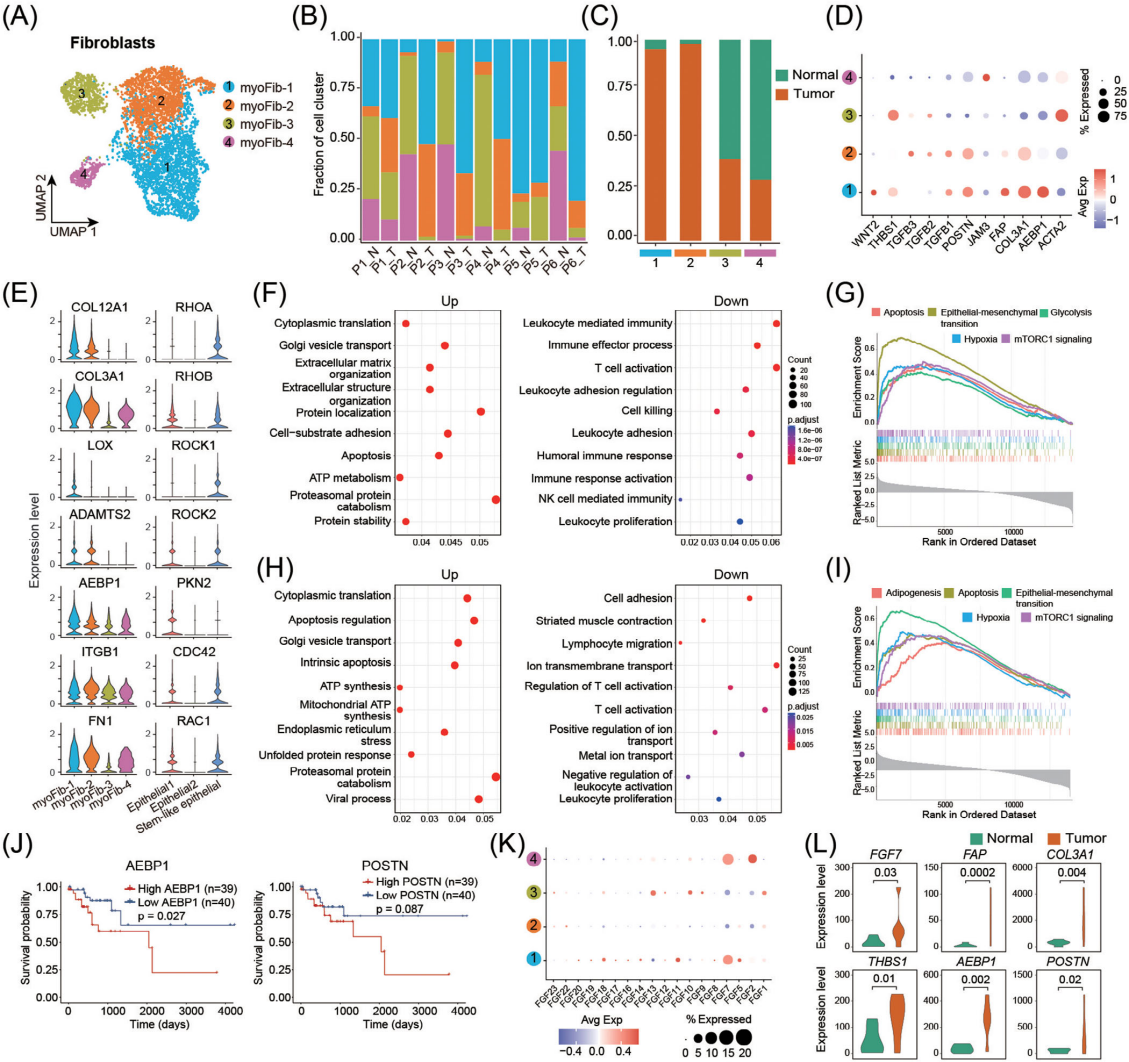
Subsets of myofibroblasts are associated with tumour progression and clinical outcome. (A) UMAP plot of myofibroblasts coloured by cell subsets. (B) Fraction of each cell subset in every sample. (C) Cell proportions of normal and tumour groups in each cell subset. (D) Expressive pattern of CAF canonical genes in each cell subset. (E) The expression level of collagen‐related genes, integrins, and fibronectin in myofibroblast subsets and Rho signalling in tumour cells. (F) Biological processes of up‐ and downregulated genes for myofib‐1. (G) Physiological processes of highly expressed genes for myofib‐1. (H). Biological processes of up‐and downregulated genes for myofib‐2. (I) Physiological processes of highly expressed genes for myofib‐2. (J) The Kaplan–Meier curves showed that MCA patients with higher expression of *AEBP1* and *POSTN* were associated with worse prognosis. (K) The graphic pattern of fibroblast growth factors in myofib‐1 and myofib‐2. (L) Expression comparison between normal and tumour groups for CAF canonical genes in myofib‐1. Green, normal group; orange, tumour group.

The dynamic remodelling of myofibroblasts in the TME suggests a functional role of these cells in MCA tumorigenesis. To thoroughly elucidate the communication between tumour cells and myofibroblasts, subpopulation analysis was conducted to identify distinct subtypes of myofibroblasts involved in MCA progression. The dimensionality reduction algorithm PCA and clustering approach UMAP were employed in the myofibroblast populations, and four subsets were presented, termed myoFib‐1 (*n* = 2387), myoFib‐2 (*n* = 1616), myoFib‐3 (*n* = 606), and myoFib‐4 (*n* = 292) (Figure 3A). The difference in myofibroblast composition between each donor's tumour and adjacent normal tissues was compared (Figure 3B, C; Figure S6A, B). It was found that myoFib‐1 and myoFib‐2 were predominantly present in tumour tissues, while myoFib‐3 and myoFib‐4 were enriched in adjacent normal tissues. Importantly, myoFib‐1 and myoFib‐2 are tumour‐specific myofibroblasts that account for 95% and 98%, respectively, in tumour tissues. Furthermore, the myoFib‐1 subset expressed the markers *FAP*, *COL3A1*, and *AEBP1*, which are typically associated with tuning cancer‐associated fibroblast activation and extracellular matrix remodelling, while myoFib‐2 showed higher expression of genes encoding growth factors, including *TGFB1*, *TGFB2*, and *TGFB3* (Figure 3D). Moreover, we further investigated the properties of CAFs for myoFib‐1 and myoFib‐2 subsets by detecting the expression of signature genes for ECM deposition and Rho signalling (Figure 3E), which are reported to play an important role in the crosstalk between CAFs, cancer cells, and tumourous physical microenvironment.^29^, ^30^, ^31^ Notably, the expression of collagen‐related genes, integrins, and fibronectin showed a significant increase in myoFib‐1 and myoFib‐2 subsets (Figure 3E). Accordingly, an expressive ascension of essential genes of Rho signalling was observed in stem‐like epithelial cells and malignant epithelial cells (Figure 3E). These results suggested that myoFib‐1 and myoFib‐2 subsets might act as CAFs and contribute to ECM deposition, which remoulds the physical environment of the tumoural niche and promotes tumour progression through integrins‐dependent fibronectin assembly and Rho signaling activation and transduction.^29^, ^30^, ^31^

To further explore the functional role of these two subsets in MCA progression, we analyzed the differentially expressed genes and performed GO enrichment and gene set enrichment analysis (Figure S6C, D). Genes highly expressed in myoFib‐1 were involved in extracellular matrix organisation and cell‐substrate adhesion, implying the involvement of these cells in ECM formation (Figure 3F, G). For myoFib‐2, highly expressed genes were mainly correlated with the processes of ATP synthesis, endoplasmic reticulum stress, and protein catabolism, suggesting a metabolism remodelling in this subset (Figure 3H, I). Importantly, leukocyte‐mediated immunity and T‐cell activation processes were both downregulated in myoFib‐1 and myoFib‐2 subsets, indicating a crucial role of these cells in the immunosuppressive TME of the MCA.

Notably, the high expression of signature genes *AEBP1* and *POSTN* in myoFib‐1 is associated with poor prognosis of MCA patients, indicating the pivotal role of myoFib‐1 in MCA progression (Figure 3J). In addition, the expression characteristics of fibroblast growth factors (FGFs) were detected in the myoFib‐1 and myoFib‐2 subsets since FGF proteins closely correlate with tumour growth and the development of colorectal cancer.^32^ Intriguingly, compared with the myoFib‐2 subgroup, myoFib‐1 displayed higher levels of *FGF* expression, with the most prevalently expressed *FGF7*, a crucial component in stimulating the proliferation of cancer cells (Figure 3K). Importantly, most canonical CAF‐associated genes and *FGF7* in myoFib‐1 subset showed higher expression levels in tumour samples than in normal tissues (Figure 3L). Moreover, tumour cells could increase *THBS1* expression significantly in fibroblasts in ex vivo co‐culture assays, indicating that *THBS1* was critical for fibroblast activation (Figure S6E).

**FIGURE 4:**
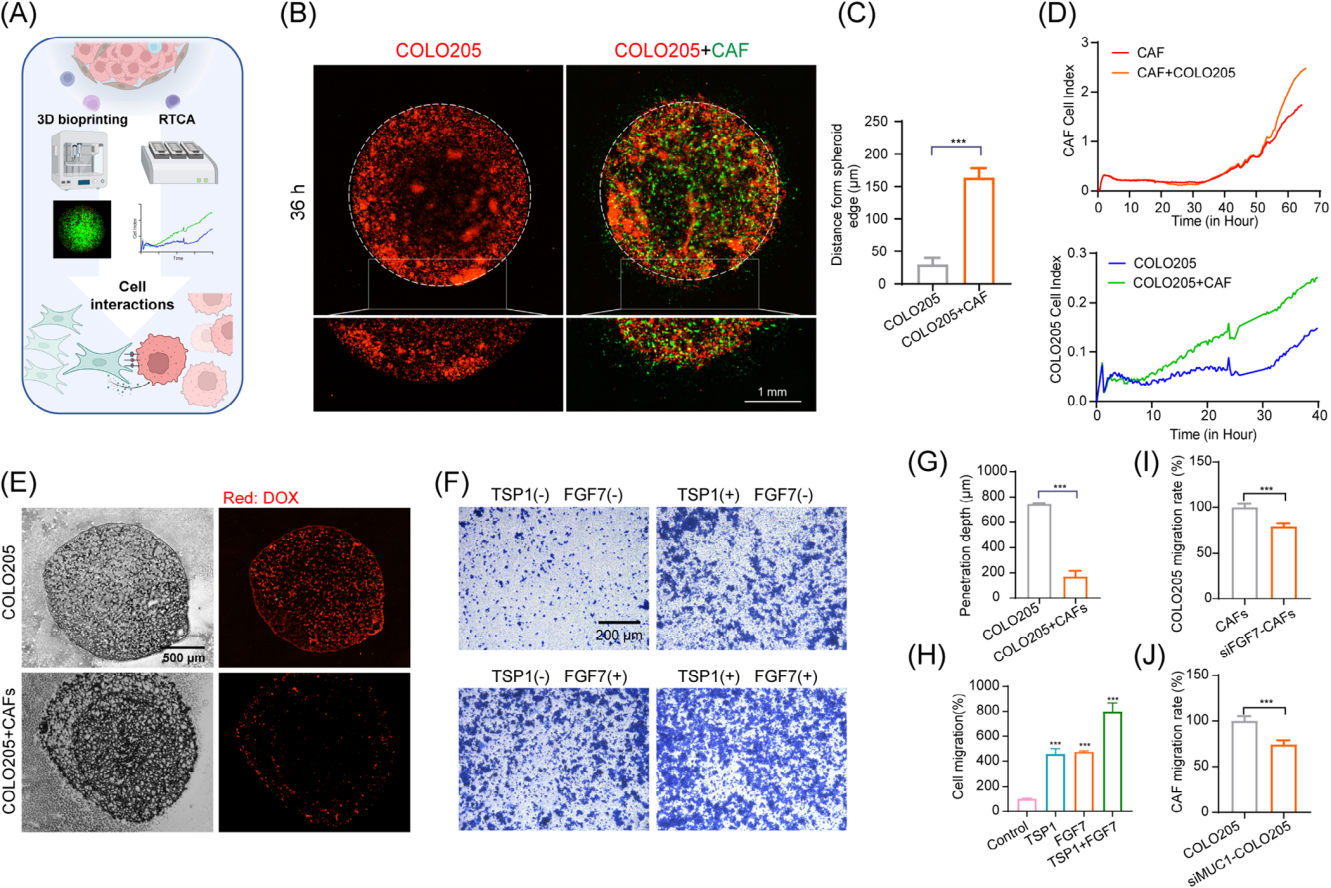
Co‐culture assay of MCA cancer cells and tumor‐associated fibroblasts. (A) Schematic illustration of the co‐culture platform. (B and C) Representative images and analysis of mCherry‐labeled COLO205 (red) cultured alone or in co‐culture with GFP‐labeled CAFs (green) and imaged by confocal microscopy after 48 h (*n* = 3). (D) Kinetic curves of COLO205 or CAFs cultured alone or in co‐culture, as assessed by xCELLigence RTCA‐DP. (E and G) Representative images and analysis of COLO205 cultured alone or in co‐culture with CAFs treated with doxorubicin for 24 h. (F and H) Transwell images and analysis of COLO205 cells cultured alone or treated with THBS1 (400 ng/mL), FGF7 (50 ng/mL), and THBS1 (400 ng/mL) in combination with FGF7 (50 ng/mL). (I) The quantitative migration rate of COLO205 when co‐cultured with CAFs or siFGF7 CAFs. (J) The quantitative migration rate of CAFs when co‐cultured with COLO205 or siMUC1 COLO205.

Subsequently, to further investigate communication between MCA cancer cells and CAFs, three classical co‐culture models were developed (Figure 4A). First, a three‐dimensional (3D) bioprinting technique was used to construct the MCA cancer cell‐CAF spheres in vitro to mimic the pathological tumour microenvironment better. It was observed that MCA cancer cells exhibited a more aggressive phenotype in the presence of CAFs, which invaded further from the culture sphere (Figure 4B, C). In addition, either MCA cancer cells or CAFs could enhance the proliferation ability of each other (Figure 4D). Wound healing assays further validated the cellular communication between cancer cells and CAFs, as the presence of CAFs augmented the migration ability of tumour cells (Figure S6F, G). Notably, co‐culture with CAFs prevented drug penetration into the tumour sphere compared with MCA cancer cell monoculture, which indicated the role of CAFs in drug resistance (Figure 4E, G). The exogenous addition of FGF7 and THBS1 protein enhanced the migration ability of cancer cells (Figure 4F, H). Moreover, we knocked down MUC1 and FGF7 in MCA cancer cells and CAFs, respectively. Either MUC1 or FGF7 knockdown impeded the migration‐promoting function of cancer cells and CAFs (Figure 4I). These results indicated that *MUC1* ^+^ tumour cells and *FGF7* ^+^ *THBS1* ^+^ CAFs exhibited significant crosstalk, promoting tumour growth and MCA progression.

### 2.5 Monocytes differentiate into macrophages in the tumoural niche and tumour‐associated macrophages contribute to immunosuppression

**FIGURE 5:**
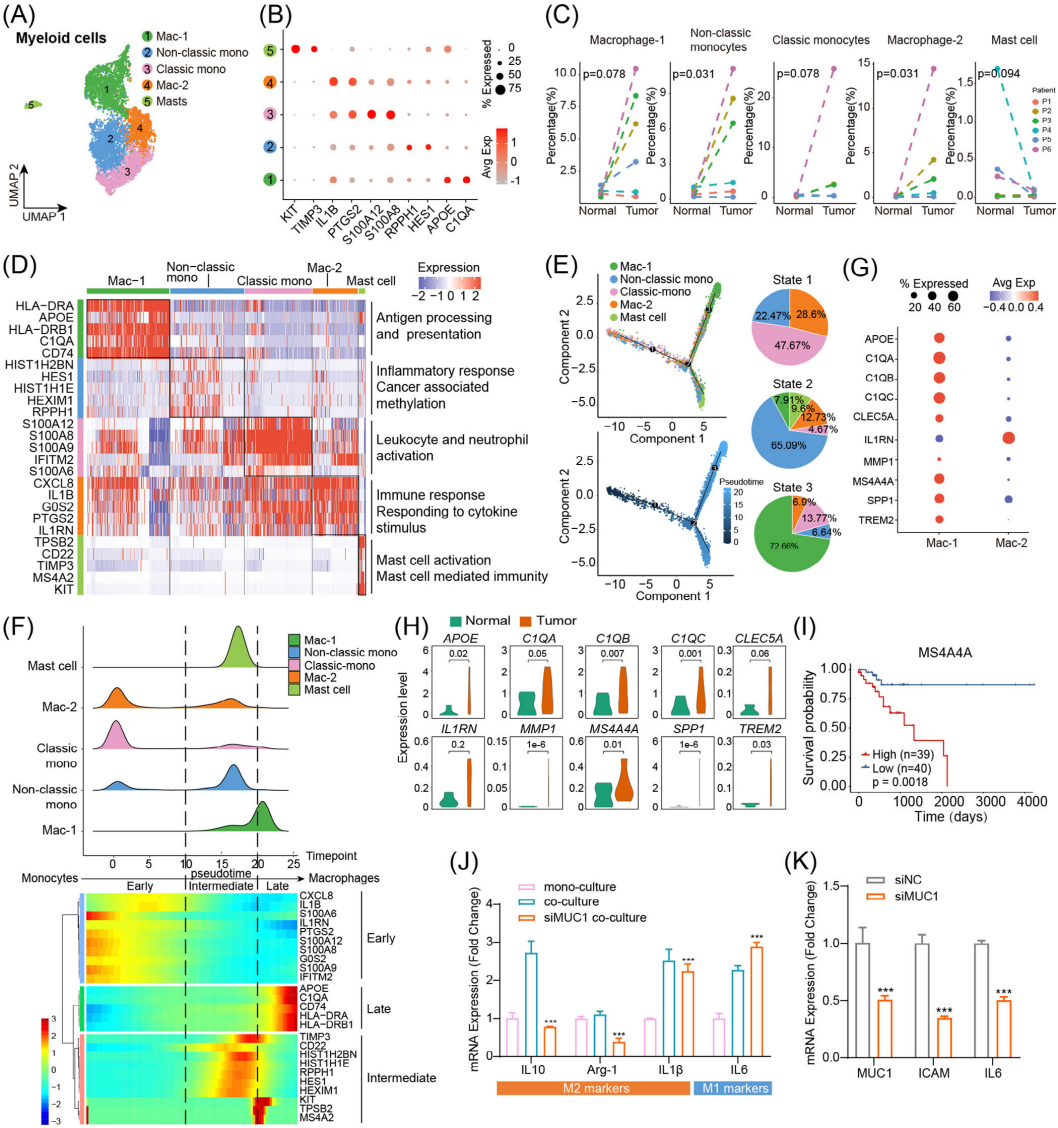
Macrophages are associated with tumour progression and immune evasion. (A) UMAP plot of myeloid cells coloured by cell subtypes. (B) Dot plots showing the expression profile of signature genes in each cell subtype. (C) Cell percentage change of each cell subtype in normal and tumour samples. (D) Heatmap showing expression profiles of feature genes in overall cells for each cell subtype. (E) Differentiation trajectory of cell subtypes. Distribution (left) and proportion (right) of each cell subtype over state of differentiation. (F) Overall gene (upper panel) and feature gene (lower panel) expression profiles of each cell subtype along with pseudotime of differentiation. (G) Expression information of tumor‐associated macrophage (TAM) canonical genes in macrophage subset 1 and 2. (H) Expression comparison of TAM canonical genes between normal and tumour samples in macrophage subset 1. (I) The Kaplan–Meier curve showed that MCA patients with higher expression of *MS4A4A* were associated with poorer prognosis. (J). qPCR analysis of M1 and M2 macrophage markers in monoculture THP1 cells, THP1 cells co‐cultured with COLO205, or THP1 cells co‐cultured with siMUC1 COLO205. (K) qPCR analysis of MUC1, ICAM, and IL6 mRNA levels in COLO205 cells after MUC1 knockdown.

Sub‐population analysis was conducted for myeloid cells since they were composed of heterogeneous populations and played complex roles in tumorigenesis. Five myeloid cell subtypes were presented, including macrophage subset 1 identified by high expression of *C1QA* and *APOE* (*n* = 2393), macrophage subset 2 characterised by positive expression of *PTGS2* and *IL1B* (*n* = 1315), classical monocytes expressing *S100A8* and *S100A12* positively (*n* = 1938), nonclassical monocytes marked by *HES1* and *RPPH1* expression (*n* = 2143), and mast cells expressing *TIMP3* and *KIT* remarkably (*n* = 188) (Figure 5A, B). Interestingly, the composition of myeloid cells changed dramatically from normal to tumour tissues. Mast cells showed a high percentage in the normal group, consistent with previous studies in which mast cells were reported to have anti‐cancer properties and inhibit tumour cell proliferation and invasion (Figure 5C and Figure S7A).^24^ We particularly value the importance of monocytes and macrophages due to their reported critical role in forming an immunosuppressive TME,^33^, ^34^ facilitating MCA progression. The cell percentage changes and cellular population diversity of monocytes and macrophages are shown in Figure 5C and Figure S7A. Monocyte and macrophage subtypes expressed different transcriptomic profiles, including enrichment of antigen processing and presentation in macrophage subset 1, responding to cytokine stimulus in macrophage subset 2, inflammatory responses in nonclassical monocytes, and leukocyte and neutrophil activation in classical monocytes (Figure 5D). Classic monocytes, macrophages (subset 2), and mast cells showed characteristics of immune cells, while non‐classic monocytes and macrophages (subset 1) presented hallmarks of tumour‐associated cells. It is well known that monocytes are direct precursors of macrophages and can differentiate into tumour‐associated macrophages in the tumoural niche.^35^ Therefore, the differentiation trajectory and pseudotime of cells of each subtype were inferred to explore the dynamics of these cells along with MCA development (Figure 5E, F). Notably, classic monocytes and macrophages (subset 2) showed more cell activity and high gene expression in state 1 of differentiation, which meant they were major cells of the early stage of tumour progression and showed immune response characteristics. Non‐classic monocytes and macrophages (subset 1) occurred in states 2 and 3 of differentiation and showed higher gene expression in the intermediate and late stages of tumorigenesis. Based on these results, we speculated that myeloid cells were remoulded in TME, and they were recruited and tamed by cancer cells to initiate the transition of monocytes to macrophages.

In addition, macrophage subset 1 expressed the *MS4A4A*, *C1QB*, *C1QC*, and *SPP1* markers typically associated with the polarisation of macrophages (Figure 5G). It was also found that macrophage subset 1 in tumour samples expressed higher M2 macrophage marker genes than those in normal tissues, indicating a tumour‐promoting role of the cells in MCA progression (Figure 5H). Previous studies have shown that MS4A4A promoted M2 polarisation of macrophages by activating the PI3K/AKT and JAK/STAT6 pathways. MS4A4A blockade treatment could reduce the infiltration of M2 macrophages and exhausted T cells, and increase effector CD8^+^ T‐cell infiltration.^36^ Prognostic analysis showed a higher expression of macrophage subset 1 signature gene *MS4A4A* was associated with worse prognosis in the TCGA‐MCA cohort (Figure 5I). Furthermore, the co‐culture assay between macrophages and MCA cell lines was conducted to verify macrophage polarisation trends in the tumour niche. We found that macrophages co‐cultured with COLO205 tumour cells exhibited an M2 phenotype, and the associated genes were also upregulated. In addition, the co‐culture supernatant from MCA cancer cells and tumour‐associated fibroblasts was also sufficient to promote the M2 polarisation of macrophages (Figure S7B). To examine the role of MUC1 in immunosuppression, we knocked down MUC1 in tumour cells and determined the expression of immune checkpoint inhibitor PD‐L1. We found that MUC1 knockdown increased PD‐L1 expression, indicating the high expression of MUC1 in tumour tissues leads to resistance to anti‐PD‐L1 immunotherapy (Figure S7C). To further explore the crosstalk between MCA tumour cells and macrophages, we knocked down MUC1 in tumour cells and then co‐cultured with macrophages. The results showed that siMUC1 tumour cells did not induce macrophage polarisation toward the M2 phenotype (Figure 5J), and MUC1 knockdown led to a decrease in the cytokines ICAM and IL6 (Figure 5K). These results demonstrated that a significantly higher percentage of *MS4A4A* ^+^ macrophages was enriched in the MCA tumour microenvironment, promoting immunosuppression through its communication with *MUC1* ^+^ tumour cells.

### 2.6 Tip cells are involved in endothelial‐to‐mesenchymal transition and are associated with tumorigenesis

**FIGURE 6:**
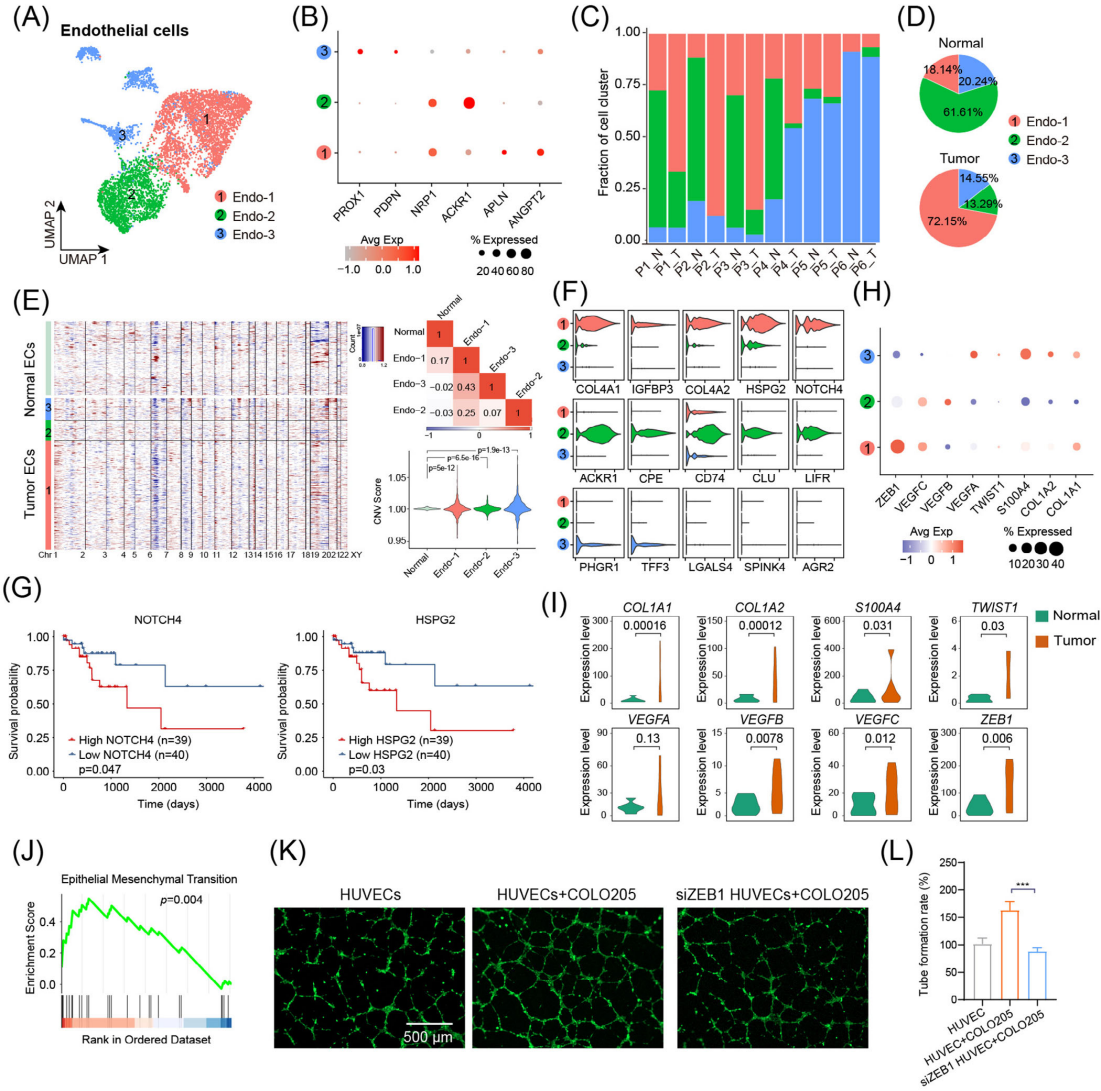
Endothelial tip cells are involved in endothelial‐to‐mesenchymal transition and are associated with tumorigenesis. (A) UMAP plot of endothelial cells coloured by cell subtypes. (B) Dot plot of signature genes in each cell subtype. (C) Fraction of each cell subtype in every sample. (D) Proportion of each cell subtype in normal and tumour groups. (E) Copy number variations (CNVs) profile in normal and tumour endothelial cells. (F) Violin plot showing the expression patterns of signature genes of each cell subtype. (G). Kaplan‐Meier curve showing differences in hazard ratios for various groups classified by the expression of key genes derived from endothelial tip cells. (H) Dot plot showing the expression profiles of EndMT‐related genes in each cell subtype. (I) Expression comparison of EndMT‐related genes between normal and tumour groups in endothelial tip cells. (J) Highly expressed genes of endothelial tip cells enriched in the process of epithelial‐to‐mesenchymal‐like transition. (K and L) Representative images and analysis of *ZEB1* knockdown on HUVEC tube formation co‐cultured with COLO205.

To explore the functions of endothelial cells (ECs) in MCA progression, subpopulation analysis was carried out to identify distinct subtypes involved in endothelial‐to‐mesenchymal transition (EndMT), an adaptive process that occurs in the tumoural niche and promotes tumour growth and dissemination.^37^ Three EC subtypes were identified: Endo‐1 (*n* = 3146), Endo‐2 (*n* = 1829), and Endo‐3 (*n* = 990) (Figure 6A). The Endo‐1 subtype was annotated as endothelial tip cells since the *APLN* and *ANGPT2* genes were highly expressed in this subtype. The Endo‐2 subtype was recognised as venous endothelial cells marked by high expression of *ACKR1* and *NRP1*. The Endo‐3 subtype was defined as lymphatic endothelial cells due to the high expression of *PDPN* and *PROX1* (Figure 6B). Endo‐1 and Endo‐3 were the dominant subtypes of tumour samples, which hinted that tip and lymphatic cells might participate in MCA tumorigenesis (Figure 6C, D; Figure S7D, E). To investigate the genomic diversity of EC subtypes, we inspected copy number variations (CNVs) of cells derived from normal and tumour samples (Figure 6E) because CNVs are hallmarks of tumorigenesis and their alterations contribute to cancer initiation, progression, and chemotherapeutic response.^38^, ^39^, ^40^ Remarkably, CNV scores of cells originating from tumour samples presented a large‐scale diversity in the whole genome compared with those from normal samples. In addition, similar CNV scores were observed in Endo‐1 and Endo‐3 subtype cells of tumour samples. These results suggested that these subtype cells might be malignant ECs and were involved in the progression of MCA.

To investigate the transcriptional profile of multiple cell subtypes, the expression patterns of each subtype's hallmark genes were detected (Figure 6F). For tip cells, we observed high expression levels of the genes *COL4A1* and *COL4A2*, which are correlated with a pro‐tumourigenic environment and promote hepatocarcinogenesis.^41^ Simultaneously, increased *NOTCH4* and *HSPG2* signals were detected, which contribute to tumour vascular structure formation.^42^ For lymphatic cells, upregulation of *PHGR1* and *TFF3* was observed, which is essential for the invasion and metastasis of colorectal cancer.^43^, ^44^ Notably, high expression of *NOTCH4* and *HSPG2* in tip cells were associated with poor prognosis of MCA patients (Figure 6G). Additionally, expression information of EndMT‐related genes, including transcription factors (*TWIST1* and *ZEB1*), mesenchymal biomarker genes (*CD44*, *S100A4*, *COL1A1*, and *COL1A2*), and vascular endothelial growth factors (*VEGFA*, *VEGFB*, and *VEGFC*), was detected for the three subtypes. Tip cells showed high expression of *ZEB1*, while lymphatic cells were highly positive for *S100A4* (Figure 6H). Notably, expression differences in EndMT genes between the tumour and normal groups were detected only in tip cells (Figure 6I). Meanwhile, gene set enrichment analysis showed that upregulated genes in tip cells presented a high enrichment score in the process of epithelial mesenchymal‐like transition (Figure 6J). These results implied that in the tumoural niche, endothelial tip cells weaken their original immune function and are tamed by tumour cells to migrate into a pre‐angiogenetic environment and trigger EndMT.^37^ The co‐culture model of COLO205 cells and human umbilical vein endothelial cells (HUVECs) further validated the crosstalk between tumour cells and endothelial cells, as the presence of tumour cells significantly enhanced the tube formation rate of endothelial cells in vitro (Figure 6K, L). Notably, the knockdown of EndMT signature gene *ZEB1* significantly abrogated the tube formation rate of HUVECs in the presence of tumour cells (Figure 6K, L), suggesting the crucial role of ZEB1 in the process of EndMT and angiogenesis.

### 2.7 Cell crosstalk between CAFs, macrophages, and tumour cells was unveiled by spatial transcriptomics

**FIGURE 7:**
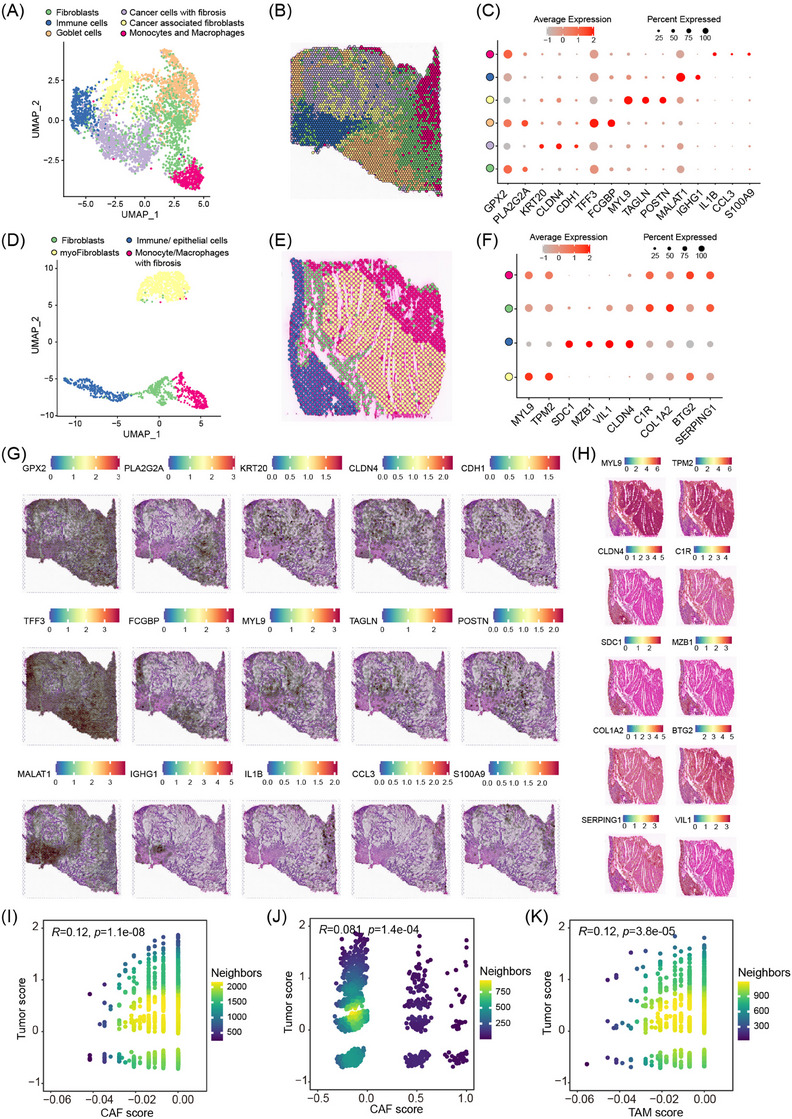
Spatial transcriptomics reveals cell crosstalk between CAFs, macrophages, and tumour cells. (A) UMAP plot of ST spots coloured by the cell type of tumour tissues from one MCA patient. (B) Clustering of ST spots and assigning a major cell type for each spot in MCA tumour tissues. (C) Dot plots of signature genes in each cell type for ST spots in tumour tissues. (D) UMAP plot of ST spots coloured by the cell type of matched normal tissues. (E) Clustering of ST spots and assigning a major cell type for each spot in normal tissues. (F) Dot plots of signature genes in each cell type for ST spots in normal tissues. (G, H) Expression and spatial distribution of signature genes in overall ST spots of tumour tissues (G) and normal tissues (H). (I) Correlation between tumour and CAF scores evaluated by the expression of the *MUC1* and *FGF7* genes. (J) Correlation between tumour and CAF scores evaluated by the expression of the *MUC1* and *THBS1* genes. (K) Correlation between tumour and TAM scores evaluated by the expression of the *MUC1* and *MS4A4A* genes.

To further verify the cell communications and crosstalk between CAFs, macrophages, and tumour cells, a spatial transcriptomics (ST) assay was conducted in tumour and adjacent normal tissues (Figure 7A, B, D, E). A transcriptome containing 3602 spots with a median depth of 854 genes was generated for the tumour tissue. Through the PCA and UMAP algorithms, 3602 spots were grouped into six clusters, where each spot was assigned as one primary cell type to investigate cell crosstalk explicitly. Notably, each spot might include several cell types since a spot of spatial transcriptomics generated by the 10x Genomics Visium platform could accommodate up to 10 cells. The six clusters were fibroblasts, marked by expression of *GPX2* and *PLA2G2A*; tumour cells with fibrosis expressed *KRT20*, *CLDN4*, and *CDH1* positively; goblet cells, identified by *TFF3* and *FCGBP* expression; cancer‐associated fibroblasts, characterised by *MYL9*, *TAGLN*, and *POSTN* expression; immune cells, expressed *MALAT1* and *IGHG1* remarkably; and monocytes and macrophages, expressed *IL1B*, *CCL3*, and *S100A9* highly (Figure 7C). The spatial distribution of signature genes for each cluster in tumour tissues is presented in Figure 7G. In addition, the spatial distribution of spots showed that tumour cells were wrapped by CAFs and fibroblasts, preventing immune cells from infiltrating into the tumour core. In addition, monocytes and macrophages were also localised around tumour cells, indicating that cell crosstalk might exist between these cells (Figure 7B). For the normal tissue, a transcriptome containing 1725 spots with a median depth of 6736 genes was detected (Figure 7D, E). A total of 1725 spots were grouped into four clusters, including myoFibroblasts, identified by high expression of *MYL9* and *TPM2*; Immune and epithelial cells simultaneously expressed signature genes of these two cells (such as *SDC1*, *MZB1*, *VIL1*, and *CLDN4*); Fibroblasts, characterised by *C1R* and *COL1A2* high expression; monocytes/macrophages with fibrosis, marked by joint expression of *BTG2* and *SERPING1* highly (Figure 7F). The spatial distribution of signature genes for each cluster in normal tissues is presented in Figure 7H. Spot spatial distribution in normal tissues showed that immune cells were inseparably intertwined with epithelial cells, which differed from tumour tissues (Figure 7E).

Since the communication between tumour cells and CAFs, unveiled by single‐cell transcriptomics, was mediated by *MUC1* and *FGF7*/*THBS1* to a certain degree, we examined the possibility of crosstalk between these two cell types in tumour tissues through expression information in spatial transcriptomics. Tumoural and CAF scores were first calculated for the union of tumour and CAF spots based on the expression signatures of the genes *MUC1* and *FGF7/THBS1*, respectively (here, fibroblasts and CAFs were combined as overall CAFs since the former might transform into the latter in tumour conditions). Then the correlative coefficient of the two scores was computed by the Spearman method to evaluate the crosstalk possibility between tumour and CAF spots (Figure 7I, J). Similarly, the possibility of crosstalk between tumour and macrophage spots was assessed by the expression signature of the *MUC1* and *MS4A4A* genes (Figure 7K). A statistically positive correlation was found in tumour‐CAF and tumour‐macrophage spots. Since a spot could include multiple cells, we speculated that tumour cells, CAFs, and macrophages were distributed in the same spots to some degree. The spatial transcriptomics analysis results confirmed that cell crosstalk existed between CAFs, macrophages, and tumour cells in the MCA.

### 2.8 Highly infiltrated *FGF7* ^+^/*THBS1* ^+^ myofibroblasts, *MS4A4A* ^+^ macrophages, and *ZEB1* ^+^ endothelial cells are associated with advanced stage and worse outcomes of MCA

**FIGURE 8:**
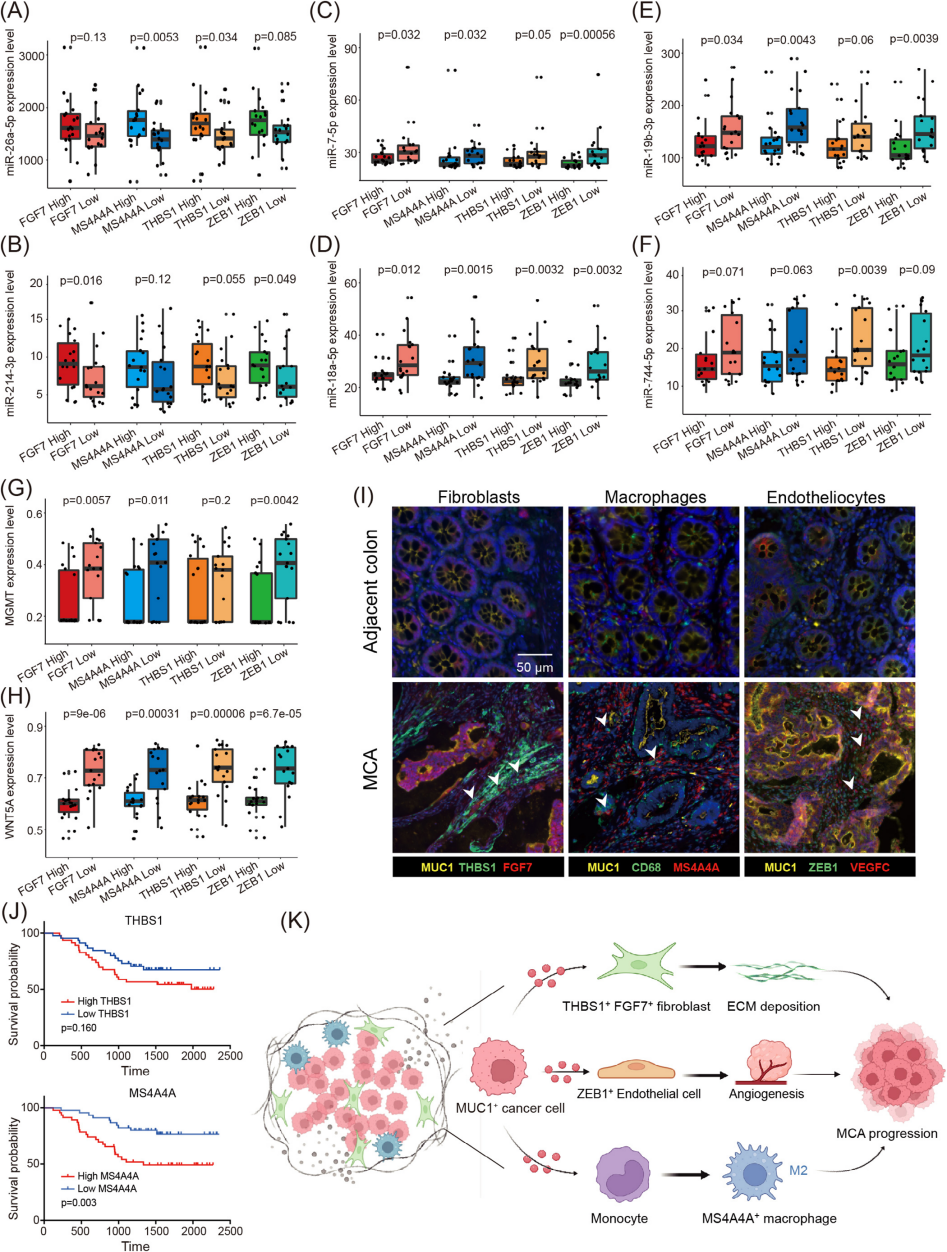
Highly infiltrated *FGF7* ^+^/*THBS1* ^+^ myofibroblasts, *MS4A4A* ^+^ macrophages, and *ZEB1* ^+^ endothelial cells correlated with an advanced stage and worse outcome of MCA. (A–F) Expressive information of miR‐26a‐5p (A), miR‐214‐3p (B), miR‐7‐5p (C), miR‐18a‐5p (D), miR‐19b‐3p (E), and miR‐744‐5p (F) in the high and low gene expression groups. (G, H). Methylation scores of the genes *MGMT* (G) and *WNT5A* (H) in the high and low gene expression groups. (I). Representative immunofluorescence staining of human MCA tissues and normal tissues. (J). Kaplan–Meier curves present the overall survival analysis for *THBS1* and *MS4A4A* in Chinese MCA patients. (K) Biological model of cell communications among tumour cells, CAFs, TAMs, and endothelial cells. The plot was created using BioRender (https://biorender.com/).

To inspect the clinical implications of cell communications between CAFs, macrophages, endothelial cells, and tumour cells, we detected expression associations between signature genes (*FGF7*/*THBS1*/*MS4A4A*/*ZEB1*) and tumour miRNAs. Emerging evidence has indicated that miRNAs can act as triggers or suppressors in colorectal cancer progression by interacting with their target genes, thus affecting tumour pathways and regulating immune response.^45^, ^46^ For example, hsa‐miR‐26a‐5p and hsa‐miR‐214‐3p are reported as triggers and are upregulated in tumour conditions.^47^, ^48^ hsa‐miR‐7‐5p, hsa‐miR‐18a‐5p, hsa‐miR‐19b‐3p, and hsa‐miR‐744‐5p are regarded as suppressors since they are downregulated in colorectal cancer patients.^49^, ^50^, ^51^ Interestingly, in the TCGA cohort (*n* = 38), MCA tumour samples with high expression of signature genes showed relatively high expression levels of hsa‐miR‐26a‐5p and hsa‐miR‐214‐3p (Figure 8A, B). Meanwhile, these samples showed lower expression of hsa‐miR‐744‐5p, hsa‐miR‐19b‐3p, hsa‐miR‐7‐5p, and hsa‐miR‐18a‐5p than signature gene low expression samples (Figure 8C–F). These results hinted that in the advanced stage of MCA (presented a high expression of *FGF7*/*THBS1*/*MS4A4A*/*ZEB1*), pro‐tumourigenic miRNAs and anti‐tumourigenic miRNAs showed increased and decreased characteristics, which regulated the activity of oncogenes and tumour suppressor genes and thus remoulded the tumour microenvironment and impacted the survival outcome of the disease.

In addition, clinical investigations show that MCA patients have a poorer chemotherapeutic response and shorter overall survival than non‐mucinous counterparts.^52^, ^53^ Therefore, we investigated whether an association existed between MCA signature genes and DNA methylation genes in response to chemotherapy. It has been reported that DNA methylation of the genes *MGMT* and *WNT5A* indicates an excellent response to 5‐FU‐based therapy in colorectal cancer.^54^, ^55^ Therefore, we detected an expression association between MCA signature genes and *MGMT* or *WNT5A* for patients in the TCGA cohort. The detection results showed that patients with high expression of signature genes had low methylation scores (Figure 8G, H), which indicated that these patients might have poor chemotherapeutic effects and worse clinical outcomes. We next performed multiplexed immunofluorescence (mIF) to quantify the spatial distribution of the selected subsets in tumour and adjacent normal tissues. We observed significantly increased infiltration of *FGF7* ^+^/*THBS1* ^+^ myofibroblasts, *MS4A4A* ^+^ macrophages, and *ZEB1* ^+^ endothelial cells in MCA tissues, broadly consistent with our scRNA‐seq data (Figure 8I). In addition, in our Chinese MCA cohort, patients with higher expression of *THBS1* ^+^ and *MS4A4A* ^+^ exhibited poorer clinical outcomes, consistent with the results of the TCGA cohort (Figure 8J). Based on these results, we hypothesised that highly infiltrated *FGF7* ^+^/*THBS1* ^+^ myofibroblasts, *MS4A4A* ^+^ macrophages, and *ZEB1* ^+^ endothelial cells reflected the advanced stage of MCA, where patients presented poor chemotherapeutic response and worse survival outcome.

## 3 DISCUSSION

MCA is a malignant cancer characterised by the presence of abundant extracellular mucin in the tissue and is more aggressive than classic adenocarcinoma.^2^ Clinical investigation shows that MCA is associated with adverse metastatic patterns, poor chemotherapeutic response, and worse survival outcomes, which warrants more studies to elucidate the pathological mechanism. In the present study, by integrating data from scRNA‐seq, spatial transcriptomics, cell co‐culture assays, immunofluorescence, and clinical outcomes of the TCGA cohort and Chinese cohort, we provided a comprehensive landscape of tumour tissues and normal counterparts for MCA at a single‐cell resolution level. Meanwhile, we elucidated cell communications between myofibroblasts, macrophages, endothelial cells, and tumour cells, as well as their clinical impacts. The results of the study facilitated the understanding of the molecular mechanism of MCA and might play an instructive role in chemotherapy and immunotherapy for MCA.

In this study, we explored the cellular landscape of the MCA tumour microenvironment by analyzing scRNA‐seq data. It was reported by the clinical investigation that MCA presented immune‐low score characteristics.^56^ Indeed, by deciphering the dynamic changes in cellular populations and cell communications between tumour and normal samples, we found that most immune cells (including naïve B cells, NK cells, CD4^+^ T cells, CD8^+^ T cells, and plasma cells) showed decreased percentages in tumour samples, which implicated immunosuppression in the tumoural niche. Importantly, we found that myofibroblasts, myeloid cells, and endothelial cells showed higher percentages in tumour samples, and they remoulded morphogenesis of the TME by regulating the expression of genes involved in the ECM correlation pathways, collagen metabolic pathways, cytokine, and chemokine association pathways, and EMT related pathways.

Afterwards, we performed cell subpopulation analysis for myofibroblasts, myeloid cells, and endothelial cells to identify distinct cell subtypes contributing to the TME. We then revealed their functions in promoting tumour cell proliferation, invasion, and immune evasion. For myofibroblasts, two subsets of this cell cluster (myoFib‐1 and myoFib‐2) were significantly upregulated in tumour tissues, and many oncogenes and CAF canonical genes showed high expression in the myoFib‐1 subset, especially the genes of *FGF7* and *THBS1*. It was demonstrated that FGF7 interacts with FGF receptor 2 (FGFR2) and promotes tumour cell invasion in colorectal cancer.^57^ THBS1 facilitates tumour cell invasion and metastasis by enhancing epithelial‐mesenchymal transition.^58^ The above evidence suggested that *FGF7* ^+^ *THBS1* ^+^ myofibroblasts play an essential role in MCA progression. Subsequently, we verified the communication between tumour cells and CAFs via cell co‐culture assays. We focused on the functions of MUC1 and FGF7 in cell communication since they showed a remarkably high expression in tumour cells and CAFs, respectively. The results of cell co‐culture and spatial transcriptome assays confirmed that MUC1 and FGF7 mediated the communication between tumour cells and CAFs to a certain degree. MUC1 is a transmembrane mucin glycoprotein expressed in tumour cells, and it is also a well‐established multifaceted oncoprotein with a critical role in tumorigenesis and may serve as a potential target for cancer vaccines.^59^, ^60^ In breast cancer, the transmembrane MUC1‐C‐terminal subunit (MUC1‐C) interacts with EGFR, ErbB2, and other receptor tyrosine kinases and contributes to the activation of the PI3K→AKT and MEK→ERK pathways.^61^ MUC1 is also associated with epithelial ovarian cancer (EOC) metastasis and the MUC1 level in blood can serve as a diagnostic biomarker for EOC diagnosis.^62^ Tumour cell derived‐MUC1 can interact with nuclear factor‐kappa B (NF‐κB) to induce the expression of interleukin‐6 (IL‐6), an essential cytokine for fibroblast activation and migration.^60^ CAFs derived‐FGF7 is a mesenchyme‐specific heparin‐binding growth factor, which can bind to FGFR2 and promote cell migration and invasion in gastric cancer by upregulating THBS1 and elevating the activity of the PI3K/Akt/mTOR signaling pathway.^63^ These findings provide profound insights into the mechanism of MCA and present potential biomarkers for diagnosing and treating MCA.

In addition, we found that monocytes might be polarised into M2‐type macrophages in co‐culture models with tumour cells, which promoted cancer cells’ evasion of immune surveillance. Moreover, co‐culture results also showed that polarisation of macrophages was mediated by MUC1 expressed by tumour cells. Generally, M1‐type macrophages act as tumour suppressors, while M2‐type macrophages are tumour triggers and promote cancer cell invasion and metastasis. As an important component of the TME, TAMs regulate multiple critical oncogenic processes and affect drug resistance in the tumoural niche.^64^, ^65^ In addition, cell communication between macrophages and tumour cells in the MCA was confirmed by spatial transcriptomics, which is consistent with some studies of CRC.^66^

The endothelial cells were classified into tip cells, vascular cells, and lymphatic cells according to marker genes. Based on the proportion of cell subtypes in the tumour group and the expression of EndMT‐related genes, we speculated that tip cells were pivotal participants in EndMT. *ZEB1* is an EndMT‐inducing transcription factor involved in cancer cell metastasis through the Akt‐MAPK and Wnt pathways.^67^ Interestingly, *ZEB1* showed a significant increase in tip cells of tumour samples, and the knockdown of *ZEB1* significantly abrogated the tube formation rate of HUVECs in the presence of tumour cells. It has been reported that angiogenesis is initiated by EndMT, which might provide a source of CAFs and induce polarisation of M2‐type macrophages.^68^, ^69^

Based on these results, we proposed a biological model of cell communication between tumour cells, CAFs, macrophages, and endothelial cells for tumorigenesis (Figure 8K). With the development of MCA, numerous MUC1 proteins were secreted by cancer cells and accumulated in the tumour microenvironment. Then, they stimulated FGF7 secretion in CAFs, which activated FGF7‐FGFR2 signalling as well as the PI3K pathway in cancer cells and induced THBS1 and TGF‐β upregulation. Increased THBS1 facilitated tumour cell invasion and metastasis. Meanwhile, elevated TGF‐β stimulated the initiation of EndMT, which was associated with angiogenesis and contributed to fibroblast activation. In addition, the high expression of MUC1 drove monocytes to differentiate into TAMs, which also promoted the migration and invasion of tumour cells. In the MCA tumoural niche, CAFs, TAMs, and endothelial cells were recruited and tamed by tumour cells. Then they boosted tumour cell proliferation, migration, invasion, and resistance to chemotherapy and immunotherapy.

In conclusion, our results provided deep insights into the molecular mechanisms of MCA, especially the complex cellular communication network in the MCA tumour microenvironment. We found that *FGF7* ^+^/*THBS1* ^+^ myofibroblasts, *MS4A4A* ^+^ macrophages, and *ZEB1* ^+^ endothelial cells were tumour‐enriched cell subtypes in MCA, contributing to MCA progression through crosstalk with *MUC1* ^+^ cancer cells. These findings facilitated the discovery of novel biomarkers for MCA diagnosis and the development of new drugs or therapies for MCA.

## 4 METHODS

### 4.1 Sample

Paired tumours and adjacent normal tissues from six MCA patients were fresh‐processed for scRNA‐seq. In practice, fresh normal mucosa and MCA tissue were washed with ice‐cold PBS and minced into small pieces. Then, the normal and tumour tissue pieces were digested with collagenase VIII at 0.38 mg/mL and DNase I at 0.1 mg/mL in 30% FBS for 1.0 h at 37°C. After digestion, tissue pieces were washed with PBS and processed according to the sample preparation instructions of the 10× Genomics platform. Moreover, one MCA tumour tissue sample and it's corresponding normal sample were fresh‐processed for spatial transcriptomics. The fresh tissue was first embedded through optical coherence tomography (OCT). Then, the tissue was cut into 10 μm sections in a cryostat and prepared based on the instructions of the Visium Spatial platform of 10x Genomics. In addition, twelve samples from six MCA patients were processed for multiplexed immunofluorescence. Three tissue microarray blocks that consisted of tumour and adjacent non‐tumour tissues from ninety MCA patients were analyzed by immunohistochemistry.

### 4.2 Multiplexed immunofluorescence

Multiplexed immunofluorescence (mIF) was performed by staining 4‐μm‐thick formalin‐fixed, paraffin‐embedded whole tissue sections with standard, primary antibodies sequentially and paired with a TSA 7‐color kit (D110071‐50T, Yuanxi Bio). The primary antibodies were MUC1 (Abcam; catalog no. ab109185; RRID:AB_10862483), THBS1 (Abcam; catalog no. ab267388), FGF7 (Sabbiotech; catalog no. 31162‐1), MS4A4A (Abcam; catalog no. ab271069), ZEB1 (Proteintech; catalog no. 21544‐1‐AP; RRID:AB_10734325), VEGFC (Proteintech; catalog no. 22601‐1‐AP; RRID:AB_2879132), and CD68 (Abcam; catalog no. ab213363; RRID:AB_2801637). After multiplexed immunofluorescence staining, each slide was treated with two drops of DAPI, washed in distilled water, and manually coverslipped. Slides were air‐dried and mounted with anti‐fade mounting medium, and pictures were taken with PANNORAMIC MIDI II. Images were analyzed using Indica Halo software.

### 4.3 Quality control of scRNA‐seq data

The raw sequencing data generated by the 10× Genomics platform were first processed with Cell Ranger software (v3.1.0) with default parameters (https://www.10xgenomics.com/; RRID:SCR_017344). The human genome (GRCh38) was used as a reference for read alignment. Then, the expression matrix of the gene expressed in each cell was calculated based on the corresponding barcode and unique molecular identifier (UMI). Subsequently, the expression matrix was imported into the Seurat (v3.1.5) package^70^ of R software (v 3.6.1, https://www.R‐project.org) for quality control with the following criteria: one gene expressed in at least three cells, one cell containing over 200 genes, and the percentage of mitochondrial genes was less than 25%. Finally, expression datasets of patient sample pairs (including the tumour sample and corresponding adjacent normal sample) were extracted from the expression matrix and normalised by the SCTransform function embedded in the Seurat package. After normalisation, these datasets were combined to generate an integrated count matrix.

### 4.4 Dimension reduction and clustering analysis for scRNA‐seq data

After quality control and normalisation, principal component analysis (PCA) was carried out for the integrated count matrix through the RunPCA function of the Seurat package. Then, the top 20 dimensions of PCA were applied in the uniform manifold approximation and projection (UMAP) algorithm provided by the Seurat package for clustering. Subsequently, the FindNeighbors and FindClusters functions embedded in the Seurat package were utilised to identify cell clusters and cell subpopulations with the resolution parameter set to 0.3 and 0.15, respectively. Finally, cell clusters were annotated by canonical gene markers manually extracted from the literature, including naïve B cells (MS4A1, IGHD, CD19), plasma cells (MZB1, JCHAIN, XBP1), myeloid cells (CD68, S100A8, S100A9, C1QC, IL1B, SPP1), enteric glial cells (S100B, NCAM1, GPM6B), epithelial cells (subset1, CLDN4, KRT20, CLDN7), epithelial cells (subset 2, DEFA6, ITPR2, PSCA), CD4^+^ T cells (CD4, KRT86, KRT81), CD8^+^ T cells (CTLA4, ANXA1, IL7R), NK cells (GNLY, NKG7, KLRD1), endothelial cells (CDH5, PECAM1, CD34), myofibroblasts (MYL9, TPM2, TPM1), crypt‐top fibroblasts (BMP5, SOX6, ENHO), inflammatory fibroblasts (DPT, CFD, CXCL12), and stem‐like epithelial cells (PTTG1, STMN1).

### 4.5 Differential expression analysis for scRNA‐seq data

Differential expression analysis was performed for each cell cluster between tumour and normal samples. In practice, a pseudo‐bulk expression profile of samples was first calculated by summing total UMI counts for all cells of a sample in a specific cell cluster.^71^ Then for each cell cluster, differentially expressed genes (DEGs) between tumour and normal samples were identified by the DESeq2 (v1.34.0) package^72^ of R software, which estimated differential gene expression based on the negative binomial distribution. Subsequently, the clusterProfiler (v4.2.2)^73^ package of R software was applied for the functional annotation of DEGs. The GO^74^ and KEGG^75^ databases were used to present detailed information on the DEGs regarding molecular functions, biological processes, cellular components, and signalling pathways. Furthermore, gene set enrichment analysis was performed for DEGs to inspect whether these genes played an important role in a certain biological process, where the clusterProfiler (v4.2.2) package was applied as an analysis tool and the hallmark gene sets in MSigDB (v7.1)^76^ were used as references.

### 4.6 Survival analysis for signature genes

The gene expression data and clinical information of the patient cohort for mucinous colorectal adenocarcinoma were first collected from the TCGA website.^77^ Then, patients were divided into high and low‐expression groups based on the median expression values of signature genes. Comparison of disease‐specific survival time between high and low expression groups was conducted by calculating the hazard ratio (HR) through the R package survminer (https://github.com/kassambara/survminer), and the Kaplan–Meier survival curve was presented by the survfit and ggsurvplot functions of the package.

### 4.7 Quantitatively characterizing cell communications for scRNA‐seq data

Communications between cell clusters were inferred by the CellChat package^78^ of R software. The ligand‐receptor interaction of the CellChatDB database was used as a reference to determine direct links between cell clusters. Subsequently, a communication network between cell clusters was constructed by aggregating intercellular links. Cell communication in a particular signalling pathway was also evaluated by summarizing the link probability of ligand‐receptor pairs involved in the pathway. Communication networks between cell clusters were assessed separately for tumour and normal samples. The communication network of tumour samples was compared with that of normal samples to identify varying intercellular links and tumour‐associated pathways under disease conditions.

### 4.8 Analysis of spatial transcriptomic data

Spatial transcriptomics (ST) slides were collected from a mucinous colorectal adenocarcinoma patient through surgical operation, including tumour tissues and corresponding normal tissues. Gene expression of capture spots for ST slides was inspected by the Visium Spatial platform of 10× Genomics with the default parameters. Quality control and genome mapping for raw sequencing reads of capture spots were conducted by Space Ranger v1.3.1, and then gene‐spot count matrices were generated. Subsequently, gene‐spot count matrices were loaded into the Seurat (v4.1.1)^79^ package of R software (v 4.1.3, https://www.R‐project.org) for filtering, normalisation, dimensionality reduction, and clustering analysis. In practice, a capture spot was filtered when fewer than 100 genes were detected. Normalisation across spots was performed by the SCTransform function. Dimensionality reduction was conducted with the top 30 principal components. Clustering analysis was carried out by the uniform manifold approximation and projection (UMAP) method. The major cell type of each spot cluster was assigned according to canonical genes expressed in the cluster. Signature genes derived from scRNA‐seq were used as features for the calculation of spot expression score through the AddModuleScore function with default parameters in the Seurat package. Finally, the possibility of crosstalk between tumour cells and cancer‐associated fibroblasts or tumour‐associated macrophages was evaluated by the spot expression score.

### 4.9 Cell differentiation trajectory analysis

To explore potential functional changes and lineage differentiation of myeloid cells, the monocle algorithm (version 2.14.0)^80^ was utilised to infer the potential differentiation trajectory of monocytes, macrophages, and mast cells. The expression data of these cell populations generated by the Seurat package were used as input of the monocle algorithm to create a CellDataSet object. Then DDRTree‐based dimension reduction and cell ordering methods were conducted with signature genes of cell populations to infer the differentiation trajectory and pseudotime of these populations.

### 4.10 Detection of copy number variations

Gene expression matrixes of endothelial cells originating from tumour and normal samples were first extracted from scRNA transcriptomics. Then the human genome annotation file (hg38) was retrieved from the GENCODE website (https://www.gencodegenes.org/). CNV scores of endothelial cell subpopulations were evaluated by the InferCNV package (v 1.15.1) of R software.

### 4.11 Statistics

Data are presented as the mean ± standard error of the means (SEM), or standard deviations (SD). All statistical analyses and graphs were done using R (v4.1.3) and GraphPad Prism (v 8.0.2). The significance threshold was set to a *p*‐value less than 0.05 unless otherwise stated.

## AUTHOR CONTRIBUTIONS

Xin Luan, Weidong Zhang, and Sanhong Liu provided direction and guidance on the whole project. Haiyang Zhou, Yiwen Shen, and Guangyong Zheng drafted the manuscript. Anqi Wang, Jing Zhang, and Hao Hu collected the samples. Guangyong Zheng and Jiayi Lin provided bioinformatics support. Xin Luan, Weidong Zhang, and Sanhong Liu reviewed the manuscript and made significant revisions. The final manuscript has been approved by all authors.

## CONFLICT OF INTEREST STATEMENT

The authors declare no conflict of interest.

## ETHICS STATEMENT

This study was reviewed and approved by the Institutional Review Board of Changzheng Hospital, Naval Medical University (2022SL062). Each patient provided written informed consent before sample collection.

## Supporting information

Supporting information

Supporting information

Supporting information

## Data Availability

The raw sequencing and processed scRNA and spatial transcriptomics data could be retrieved from the Gene Expression Omnibus (GEO) database through the accession number GSE236698.
