# Supplementary material for: Integrating single‐cell and spatial analysis reveals MUC1‐mediated cellular crosstalk in mucinous colorectal adenocarcinoma
Source: Clin Transl Med. 2024 May 22;14(5):e1701. doi: 10.1002/ctm2.1701 (PMC11111627; doi:10.1002/ctm2.1701)
Supplement: Supplementary file 1 — Supporting information [file CTM2-14-e1701-s001.docx]

**Supplementary information**

**Integrating single-cell and spatial analysis reveals MUC1-mediated cellular crosstalk in mucinous colorectal adenocarcinoma**

Haiyang Zhou^1^^,2^, Yiwen Shen^1^, Guangyong Zheng^1^, Beibei Zhang^3^, Anqi Wang^2^, Jing Zhang^6^, Hao Hu^7^, Jiayi Lin^1^, Sanhong Liu^1,*^, Xin Luan^1,*^, and Weidong Zhang^1,4,5,*^

^1^Shanghai Frontiers Science Center of TCM Chemical Biology, Institute of Interdisciplinary Integrative Medicine Research, Shanghai University of Traditional Chinese Medicine, Shanghai 201203, China.

^2^Department of Colorectal Surgery, Changzheng Hospital, Naval Medical University, Shanghai 200003, China.

^3^Department of Dermatology, Tongren Hospital, Shanghai Jiao Tong University School of Medicine, Shanghai 200003, China.

^4^Institute of Medicinal Plant Development, Chinese Academy of Medical Sciences and Peking Union Medical College, Beijing 100193, China.

^5^School of Pharmacy, Naval Medical University, Shanghai 200433, China.

^6^Department of Pathology, Changzheng Hospital, Naval Medical University, Shanghai 200003, China.

^7^Department of Pathology, Changhai Hospital, Naval Medical University, Shanghai 200433, China.

Haiyang Zhou, Yiwen Shen, Guangyong Zheng and Beibei Zhang contributed equally to this work.

**Correspondence**: Weidong Zhang, Xin Luan, and Sanhong Liu, Shanghai Frontiers Science Center of TCM Chemical Biology, Institute of Interdisciplinary Integrative Medicine Research, Shanghai Univrersity of Traditional Chinese Medicine, Shanghai 201203, China.

Email: wdzhangy@hotmail.com; luanxin@shutcm.edu.cn and liush@shutcm.edu.cn

**Supplementary methods**

**4.12 Cell lines and cell culture**

The human colorectal cancer cell line COLO205 was obtained from American Type Culture Collection (ATCC) and cultured in endotoxin-free RPMI 1640 supplemented with 10% fetal bovine serum (FBS). Cancer-associated fibroblast cells were cultured using the outgrowth method in DMEM/F12 (1:1) medium supplemented with 10% FBS. All cells were cultured in a humidified incubator at 37°C and 5% CO_2_.

**4.13 Transwell migration assay**

The migration assays were performed in 24-well cultured plates with eight μm pore-size Transwell chamber inserts (Corning). To determine CAF-induced cancer cell migration, CAFs (3×10^4^ cells/well) were added to the bottom chambers of 24-well culture plates in a serum-free medium. COLO205 cells were seeded into the upper chamber of Transwell inserts at a density of 1.5×10^5^ cells/400 μl. The negative control group was treated with 1640 basic medium in the bottom chamber. To determine cancer cell-induced CAF migration, CAFs (1.5×10^5^ cells/well) were placed in the upper chamber, and COLO205 cells (3×10^4^ cells/well) were placed in the bottom chamber. After incubation at 37°C/5% CO_2_ for 24 hours, the non-migrated cells remaining on the membrane's upper surface were scraped. The migrated cells on the lower face of the membrane were fixed with 4% paraformaldehyde and stained with crystal violet, and five random fields were counted under a light microscope. Each experiment was repeated three times.

**4.14 Real-Time Cellular Analysis**

The xCELLigence RTCA systems (Agilent, USA), based on electrical impedance, allow real-time and label-free measurement of cell proliferation. Moreover, the obtained cell index reflects a comprehensive characterization, including cell adhesion and spreading. For the co-culture assay, 5×10^3^ CAFs per well were seeded on E-Plate 16 and incubated overnight. Then 1×10^4^ COLO205 cells seeded on the insert chamber were added into the E-Plate 16. Cell growth was detected by xCELLigence RTCA systems, and cell index values were calculated using RTCA software.

**4.15 3D invasion assay**

Collagen was prepared by mixing 10X M199 growth medium (1/10 final volume), 2 mg/ml rat-tail collagen type I (Corning) and growth media or conditioned media. NaOH (1 N) was added slowly while mixing to bring the pH to pH 7.3 to obtain a neutralized collagen solution. Then, 250 μL collagen was added to each well of a 24-well plate to cover the entire bottom. The plates were left in the hood until the collagen was solidified. Tumor cells alone (1×10^5^) or tumor cells with CAFs (6:1) were mixed with five microliters of collagen and the drop was placed in the center of each well. The plates were reversed until the droplets polymerized to obtain a sharp margin of the drop. Then another 250 μL of collagen was added to each well to cover the droplet. After the collagen was solidified, 500 μL of growth media was added to each well. Stitched images were taken with a Leica confocal microscope every day for five days, and invasion outside of the collagen droplet was measured with ImageJ software.

**4.16 Scratch wound healing assay**

The motility of cancer cells alone or co-cultured with CAFs was evaluated using the scratch wound healing assay. COLO205 cells (mCherry-transfected cells) alone or with CAFs (GFP-transfected cells) were seeded in a 12-well plate until confluent, wounded with a plastic pipette tip, rinsed with PBS, and incubated with growth media. Images were captured at 0 h, 24 h, and 36 h using Cytation5 (BioTek). The experiments were carried out in triplicate.

**4.17 Drug penetration studies with 3D stroma-rich spheroids**

3D stroma-rich spheroids containing COLO205 and CAFs were generated by an improved hanging drop method. Briefly, the tumor cells (1×10^5^) were mixed with 5 μl of the collagen mixture and were dropped in the center of each well. The plates were inverted until the droplets polymerized to obtain the sharp margins of the droplets. Then another 5 μl of the collagen mixture with 1×10^5^ CAFs was dropped on the tumor cell droplets. Hanging drop cultures were incubated for 3 days before 3D spheroids was harvested for penetration studies. Spheroids were fixed with 4% paraformaldehyde and exposed to 3 μM doxorubicin for 6 h, and cryosections were visualized by confocal microscopy.

**4.18 siRNA transfection**

For siRNA-mediated knockdown experiments, cells were plated in 6-well plates before transfection at an initial confluence of 20-30%. siRNAs were designed and synthesized with the following sequences: human MUC1: 5’- GAAGCAGCCUCUCGAUAUATT-3’; human FGF7: 5’-GGGCACUAUAUCUCUAGCUTT-3’; human ZEB1: 5’-CUCUGAAAGAACACAUUAATT-3’ (GenePharma). siRNAs and TransMate reagents were prepared and added according to the manufacturer’s instructions (GenePharma). siRNAs were added to the cells and transfected for 48 h.

**4.19 RNA isolation and RT-PCR**

RNA isolation from cultured cell lines was extracted with TRIzol reagent (Sangon Biotech) and immediately stored at -80°C. To perform RT-PCR, cDNA was first obtained by RNA reverse transcription using HiScript II Q RT SuperMix for qPCR (Vazyme) with 200 ng input RNA per sample. The primers were purchased from Sangon Biotech. RT-PCR was performed using ChamQ Universal SYBR qPCR Master Mix (Vazyme) and detected with a Quantstudio 3 RT-PCR system. Gene expression was determined by the threshold cycle (Ct) values normalized against the housekeeping gene. The primer sequences are listed as follows:

human MUC1:

Forward 5’-GGCCUCUCCAAUAUUAAGUTT-3’;

Reverse 5’-ACUUAAUAUUGGAGAGGCCTT-3’.

human FGF7:

Forward 5’-GATCATGCTTCCACCTCGTCTGTC-3’;

Reverse 5’-AGTTCACACTCGTAGCCGTTTGC-3.

**Supplementary Figures**


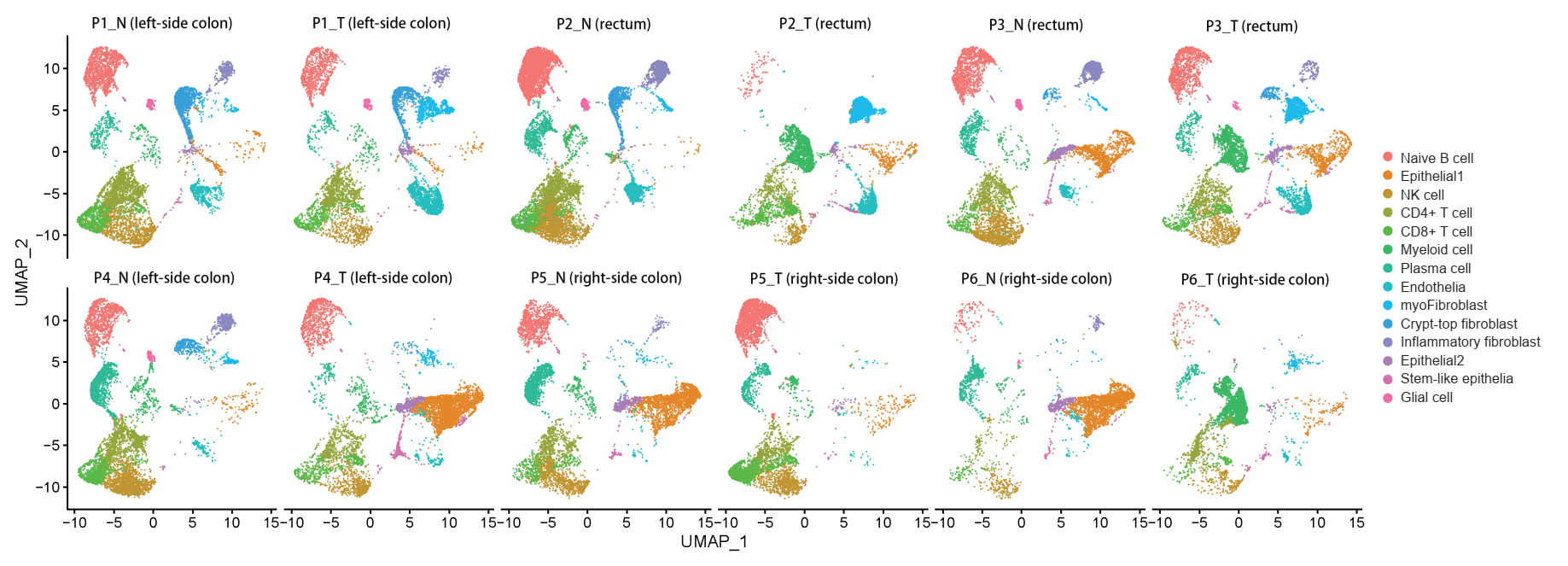


**Figure S1. Composition similarity of cell clusters in six MCA patients.** UMAP plots of fourteen cell clusters originating from tumor and normal samples of six MCA patients.


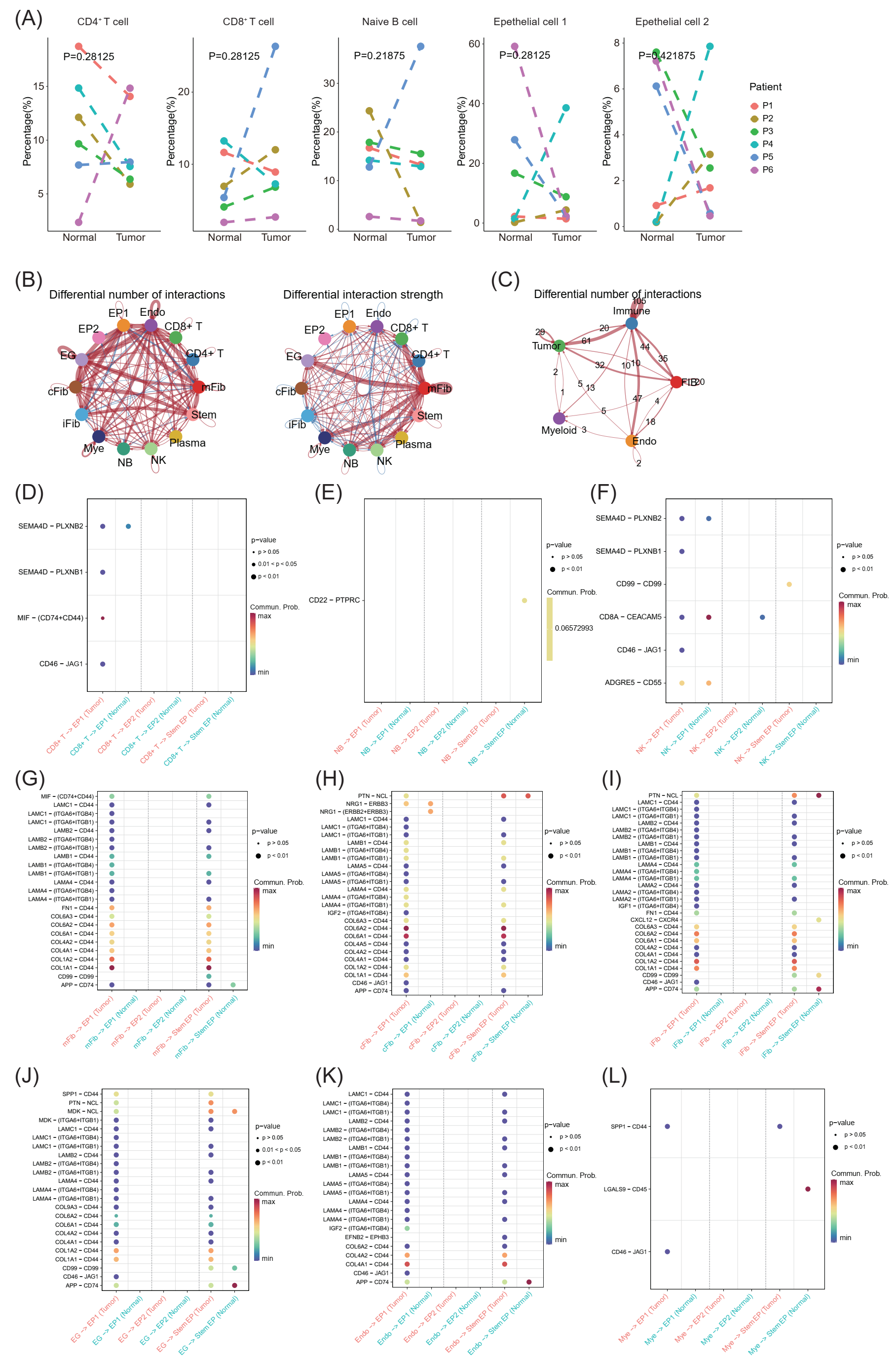


**Figure S2. Differences in communication among cell clusters between the normal and tumor groups**

**(A).** Percentage comparison between normal and tumor groups in CD4^+^ T cells, CD8^+^ T cells, naïve B cells, and epithelial cell subsets 1 and 2. **(B).** Number and strength difference of communication among cell clusters in the normal and tumor groups. **(C).** Number differences in communication among immune, fibroblast, myeloid, endothelium, and tumor modules. **(D)-(L).** Differences in ligand-receptor pair interactions in signaling pathways among cell clusters between the normal and tumor groups. **(D).** Interactions of CD8^+^ T cells and epithelial cells. **(E).** Interactions of naïve B cells and epithelial cells. **(F).** Interactions of NK cells and epithelial cells. **(G).** Interactions of myofibroblasts and epithelial cells. **(H).** Interactions of crypt-top fibroblasts and epithelial cells. **(I).** Interactions of inflammatory fibroblasts and epithelial cells. **(J).** Interactions of enteric glial cells and epithelial cells. **(K).** Interactions of endothelial cells and epithelial cells. **(L).** Interactions of myeloid cells and epithelial cells.


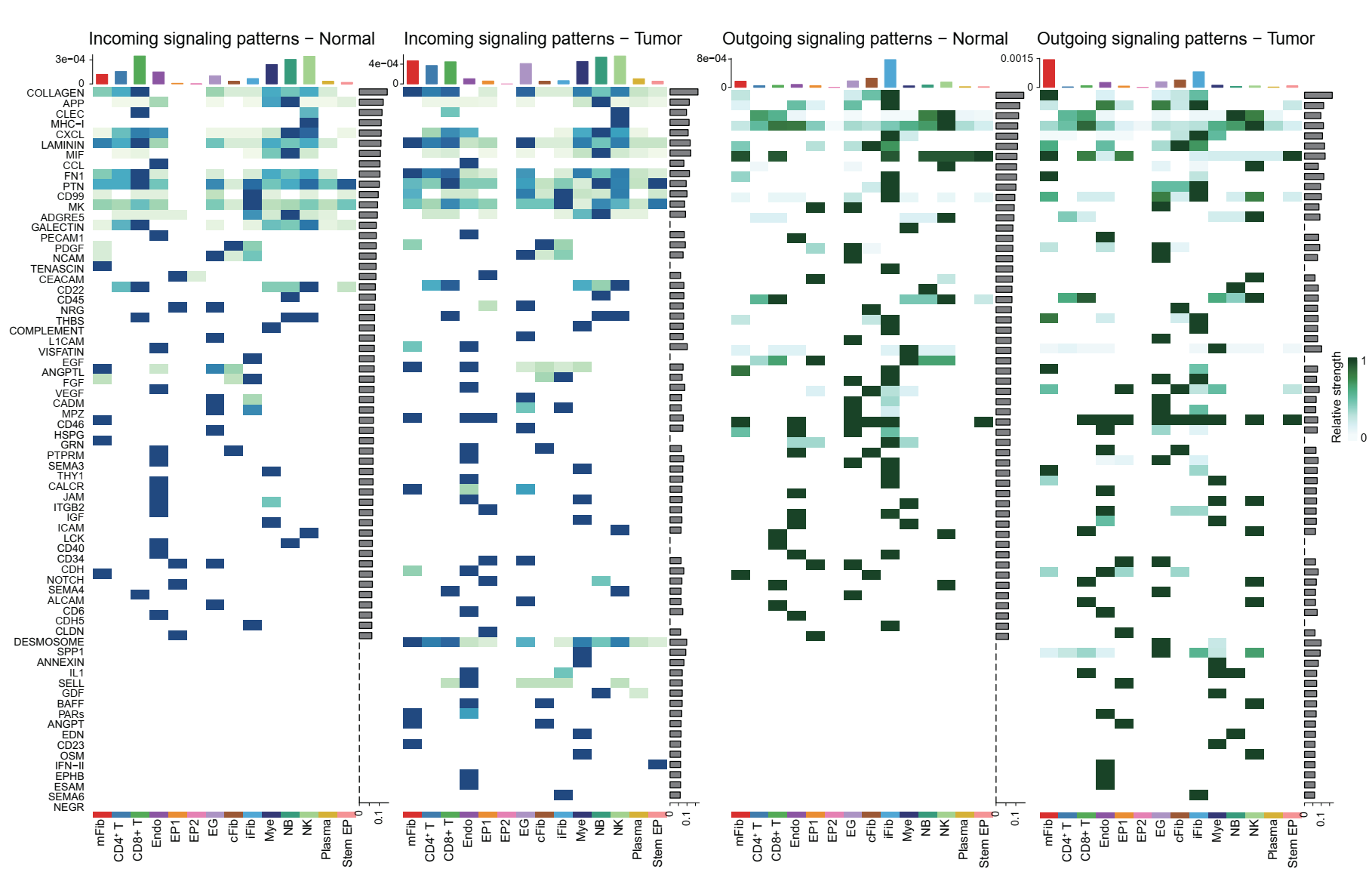


**Figure S3. Comparison of ligand-receptor pair interactions between the normal and tumor groups at the pathway level among cell clusters.**


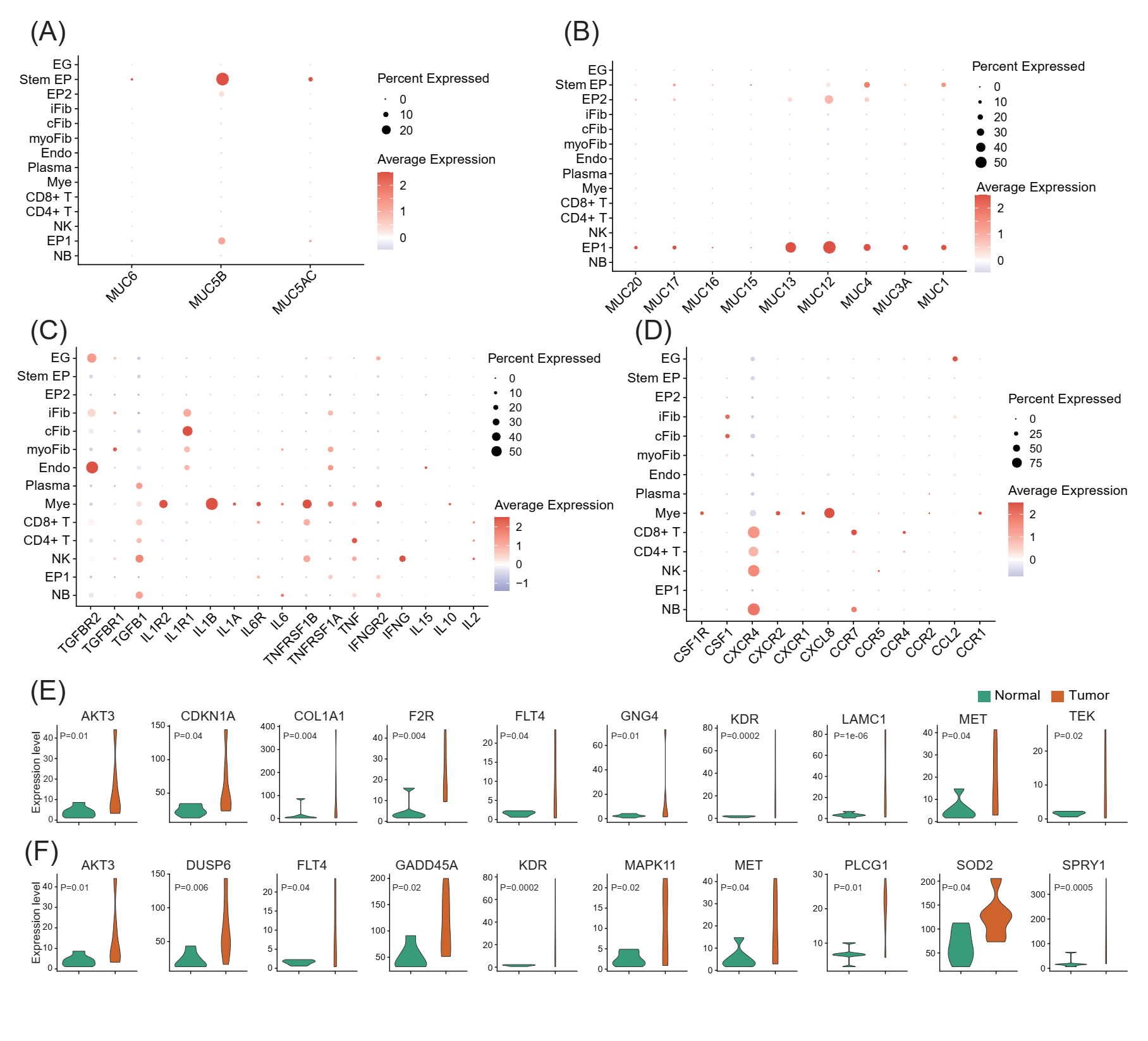


**Figure S4. Expressive information on the mucin protein family, cytokines, and chemokines in various cell clusters and the signaling pathways enriched in malignant cells**

**(A).** Expressive information of secreted mucins in each cell cluster. **(B).** Expressive information of transmembrane mucins in each cell cluster. **(C).** Expressive information of cytokines in each cell cluster. **(D).** Expressive information of chemokine receptors in each cell cluster. **(E).** PI3K-AKT signaling pathway associated genes were highly expressed in stem-like epithelial cells of tumor samples than that of normal samples. **(F).** MEK-ERK signaling pathway associated genes were highly expressed in stem-like epithelial cells of tumor samples than that of normal samples.


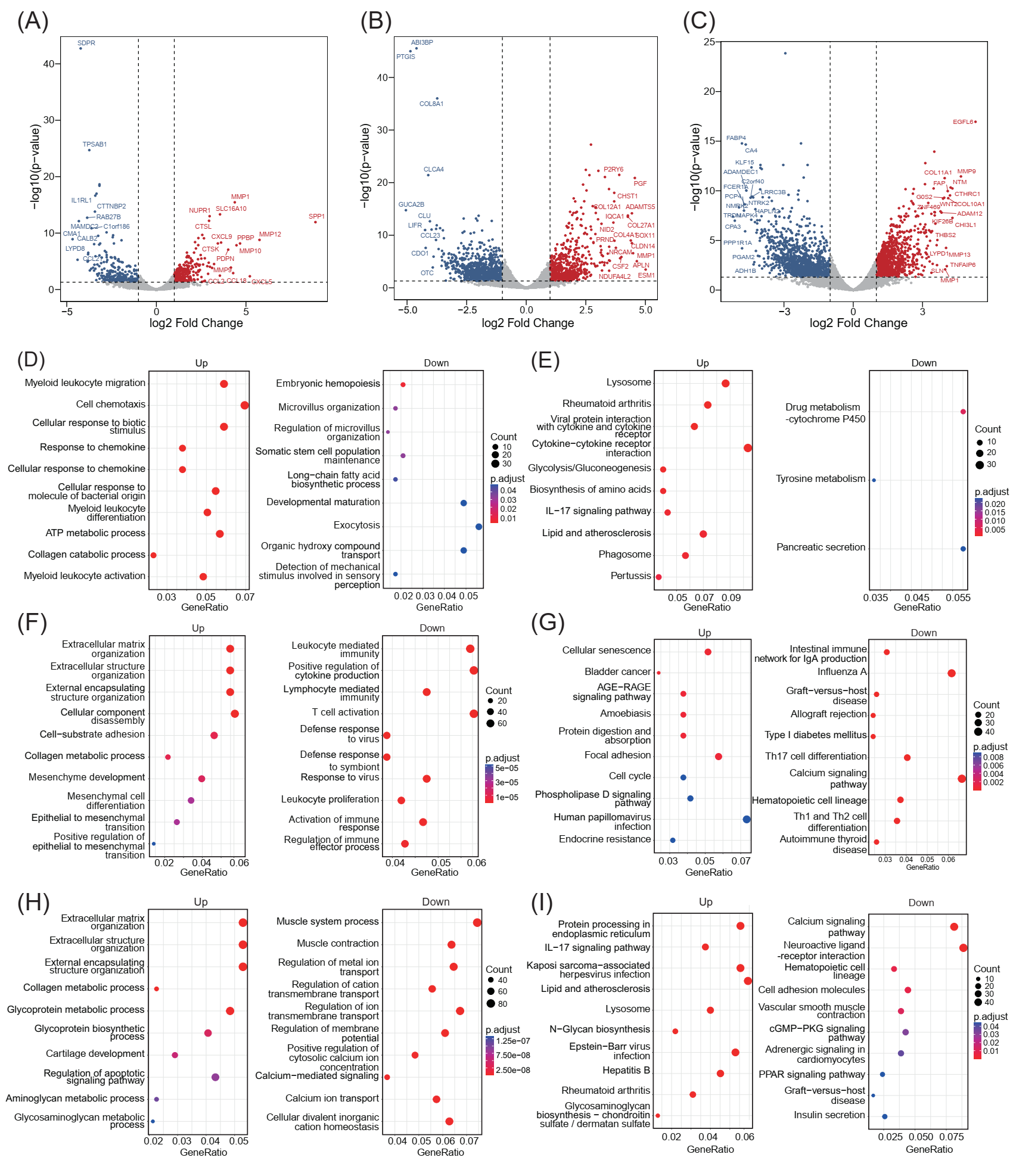


**Figure S5. Characterization of differentially expressed genes for myeloid cells, endothelial cells, and myofibroblasts between the normal and tumor groups**

**(A).** The volcano plot presents differentially expressed genes in myeloid cells. **(B).** The volcano plot presents differentially expressed genes in endothelial cells. **(C).** The volcano plot presents differentially expressed genes in myofibroblasts. **(D).** Biological processes of up- and downregulated genes for myeloid cells. **(E).** Signaling pathways of up- and downregulated genes for myeloid cells. **(F).** Biological processes of up- and downregulated genes for endothelial cells. **(G).** Signaling pathways of up- and downregulated genes for endothelial cells. **(H).** Biological processes of up- and downregulated genes for myofibroblasts. **(I).** Signaling pathways of up- and downregulated genes for myofibroblasts.


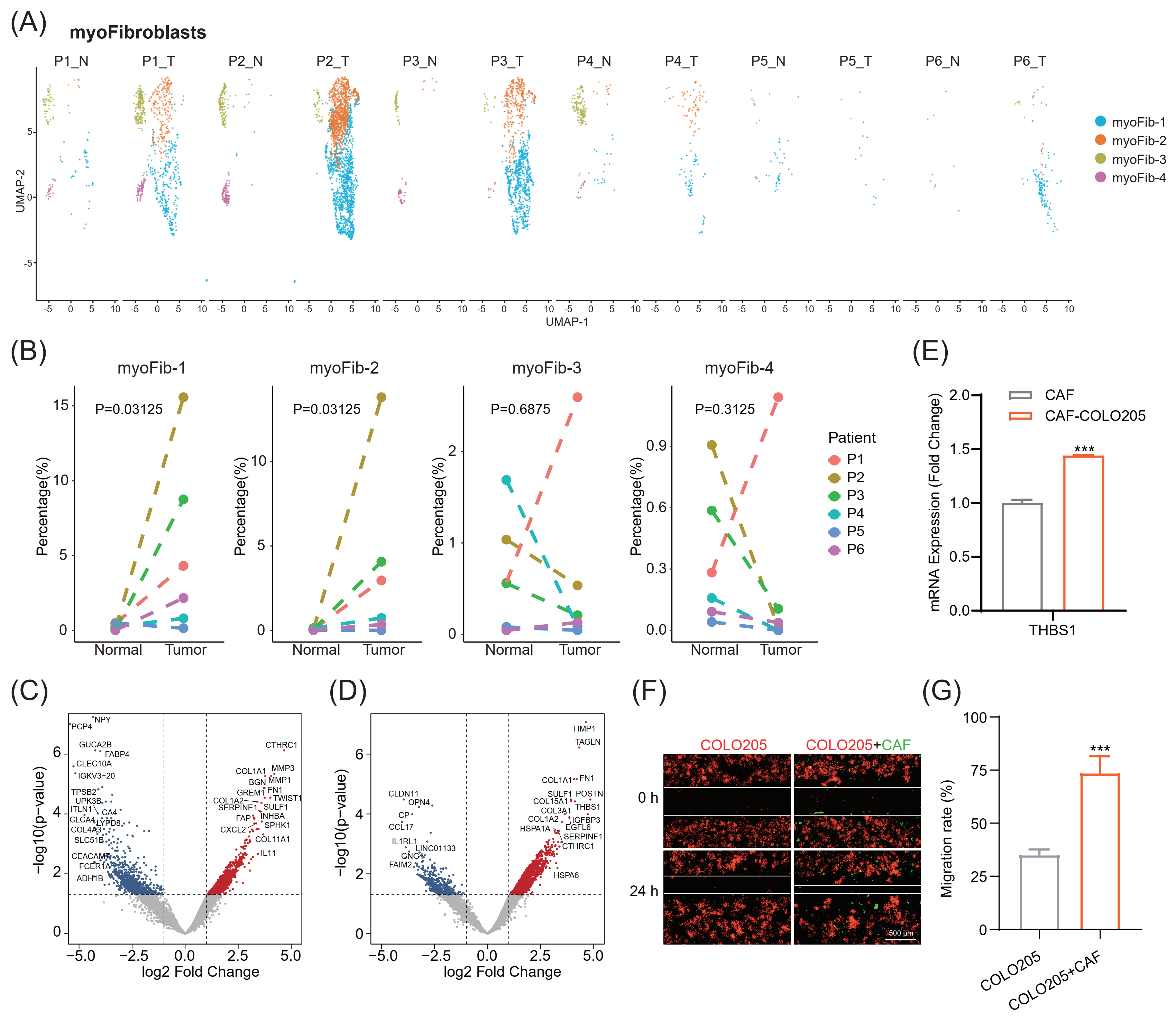


**Figure S6. Cell properties of subsets of myofibroblasts and cell co-culture characteristics of CAFs and tumor cells**

**(A).** Cellular population diversity of myofibroblasts for every sample. **(B).** Percentage comparison between normal and tumor groups in each subset of myofibroblasts. **(C).** The volcano plot presents differentially expressed genes in myofib-1. **(D).** The volcano plot presents differentially expressed genes in myofib-2. **(E).** mRNA expression level of THBS1 in CAFs with and without COLO205. **(F).** Representative images of wound healing of COLO205 (red) cultured alone or in co-culture CAFs (green). **(G).** Quantitative wound healing rate of COLO205 cells cultured alone or in co-culture CAFs.


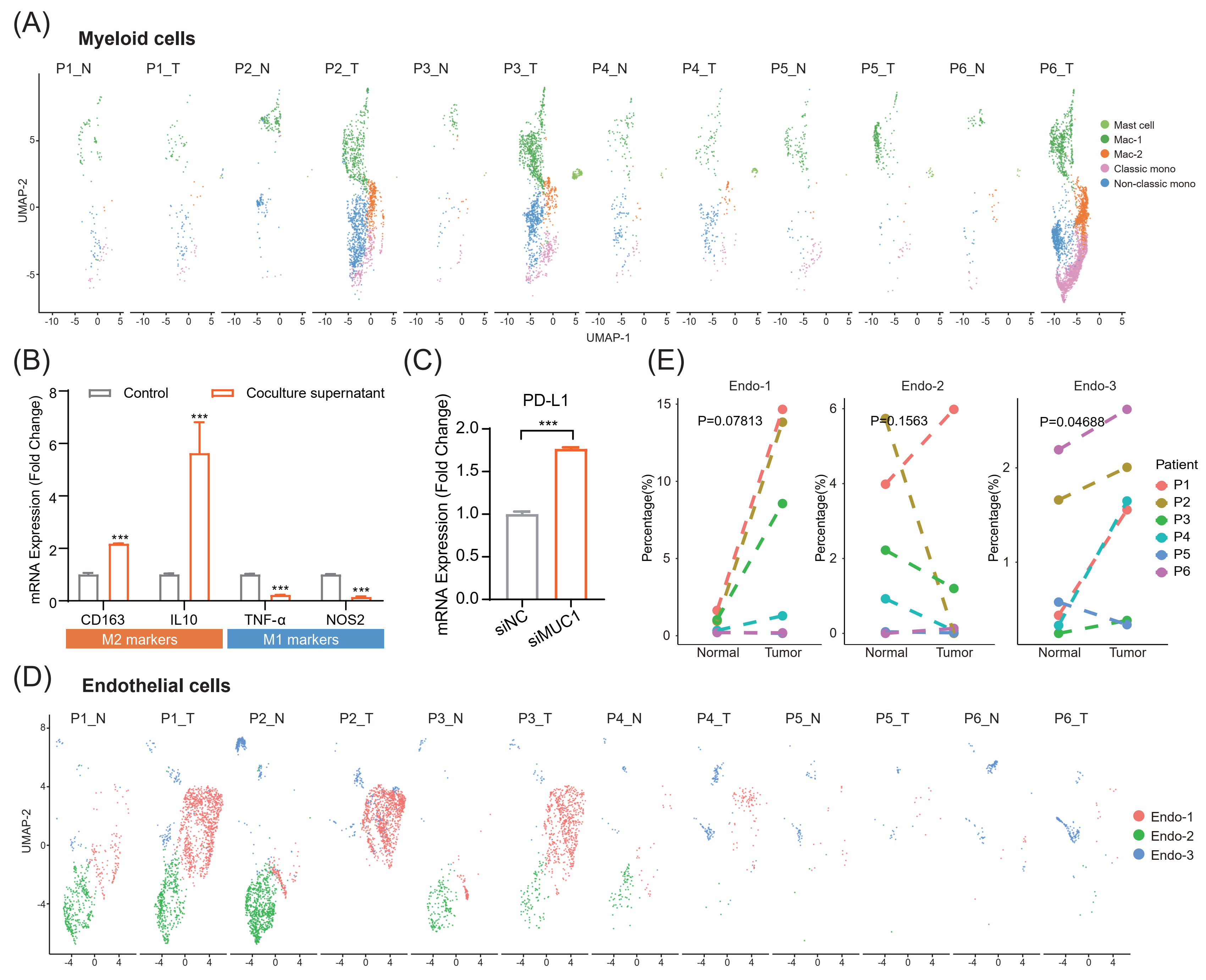


**Figure S7. Cell properties of subsets of myeloid cells and endothelial cells**

**(A).** Cellular population diversity of myeloid cells for every sample. **(B).** The coculture supernatant from MCA cancer cells and tumor associated fibroblasts could promote M2 polarization of macrophages. **(C).** MUC1 knockdown increased PD-L1 expression in COLO205 cells. **(D).** Cellular population diversity of each subtype of endothelial cells for every sample. **(E).** Percentage comparison between normal and tumor groups in each subtype of endothelial cells.
