# Supplementary material for: Integrating single‐cell and spatial analysis reveals MUC1‐mediated cellular crosstalk in mucinous colorectal adenocarcinoma
Source: Clin Transl Med. 2024 May 22;14(5):e1701. doi: 10.1002/ctm2.1701 (PMC11111627; doi:10.1002/ctm2.1701)
Supplement: Supplementary file 2 — Supporting information [file CTM2-14-e1701-s003.docx]

**Table S1: Characteristics of patients included in this study.**

| Case No. | ID | Gender | Age | Tumor location | Histology | Stage | Tumor differentiation | MSI status | Kras | Nras | Braf | Cancer recurrence | Died of cancer | Follow-up duration (days) |
| --- | --- | --- | --- | --- | --- | --- | --- | --- | --- | --- | --- | --- | --- | --- |
| 1 | CZZRJ | Male | 19 | Descending colon | Mucous adenocarcinoma | IV | Low | MSS | Wild | Wild | Wild | No | No | 625 |
| 2 | CZYMF | Female | 90 | Rectum | Mucous adenocarcinoma | III | Low | MSS | Mutant | Wild | Wild | No | No | 554 |
| 3 | CZTQH | Male | 70 | Rectum | Mucous adenocarcinoma | III | Low | MSS | Wild | Wild | Wild | No | No | 407 |
| 4 | CZXLB | Male | 69 | Sigmoid colon | Mucous adenocarcinoma | III | Low | MSS | Mutant | Wild | Wild | No | No | 379 |
| 5 | CZKJ | Male | 30 | Ileocecal | Mucous adenocarcinoma | III | Low | MSS | Wild | Wild | Wild | Yes | No | 163 |
| 6 | CZWTB | Male | 55 | Ascending colon | Mucous adenocarcinoma | II | Low | MSS | Wild | Wild | Wild | No | No | 147 |
